# Detection Bias in EHR-Based Research on Clinical Exposures and Dementia

**DOI:** 10.1001/jamanetworkopen.2025.6637

**Published:** 2025-04-23

**Authors:** Jingxuan Wang, Minhyuk Choi, Peter Buto, J. Daniel Kelly, Renaud La Joie, John Kornak, Scott C. Zimmerman, Ruijia Chen, Eva Raphael, Catherine A. Schaefer, Deborah Blacker, M. Maria Glymour

**Affiliations:** 1 Department of Epidemiology and Biostatistics, University of California, San Francisco; 2 Department of Epidemiology, Boston University, Boston, Massachusetts; 3 F.I. Proctor Foundation, University of California, San Francisco; 4 Memory and Aging Center, Department of Neurology, Weill Institute for Neurosciences, University of California, San Francisco; 5 Department of Family and Community Medicine, University of California, San Francisco; 6 Kaiser Permanente Division of Research, Pleasanton, California; 7 Department of Psychiatry, Massachusetts General Hospital, Charlestown, Massachusetts; 8 Department of Psychiatry, Harvard Medical School, Boston, Massachusetts; 9 Department of Epidemiology, Harvard T.H. Chan School of Public Health, Boston, Massachusetts

## Abstract

**Importance:**

Detection bias occurs when an exposure is associated with a systematic difference in outcome ascertainment or diagnosis. For dementia research, diagnosed health conditions that bring patients into frequent interaction with health care may increase the chance that an individual receives a dementia diagnosis.

**Objective:**

To evaluate potential detection bias or misdiagnosis bias in evaluation of clinical factors associated with dementia using electronic health record (EHR) data.

**Design, Setting, and Participants:**

This prospective cohort study used data from 2 population-based volunteer cohorts: UK Biobank (UKB) and All of Us (AOU). Participants were aged 55 years or older, were dementia-free at baseline, and had linked EHRs. Participants in UKB were followed up from baseline (2006-2010) until December 2022, and in AOU, from baseline (2017-2022) until July 2022. Data were analyzed from November 2023 through February 2025.

**Exposures:**

Diagnoses of type 2 diabetes, depression, hypertension, urinary tract infection, kidney stones, forearm fracture, and gastrointestinal (GI) bleeding.

**Main Outcomes and Measures:**

Rate of incident all-cause dementia diagnosis from EHRs and associations between clinical exposures and incident dementia diagnosis, assessed using Cox proportional hazards regression models.

**Results:**

Among 228 392 participants from UKB (n = 137 374; mean [SD] age at baseline, 62.5 [4.1] years; 53.8% female) and AOU (n = 91 018; mean [SD] age at baseline, 66.9 [7.8] years; 57.1% female), those with a history of a clinical exposure at baseline had higher dementia incidence rates compared with those without such history. For example, among participants with a history of GI bleeding, the dementia incidence rates were 3.0 (UKB) and 7.7 (AOU) per 1000 person-years compared with 2.2 (UKB) and 2.4 (AOU) per 1000 person-years among those without a history of GI bleeding. All exposures were significantly associated with incident dementia, with hazard ratios (HRs) ranging from 1.18 (95% CI, 1.00-1.40) to 3.51 (95% CI, 3.08-4.01). Risk of incident dementia was typically highest in the first year following exposure diagnosis and attenuated thereafter. For example, in the first year after GI bleeding, there were larger elevations in risk of incident dementia (HR, 2.17 [95% CI, 1.46-3.22] in UKB; HR, 2.56 [95% CI, 1.62-4.04] in AOU) compared with 1 to 5 years after bleeding (HR, 1.46 [95% CI, 1.15-1.86] in UKB; HR, 2.14 [95% CI, 1.63-2.81] in AOU).

**Conclusions and Relevance:**

In this cohort study of 2 large datasets, diagnoses of several conditions associated with varying risks of dementia were associated with a higher short-term likelihood of dementia diagnosis. This finding suggests that diagnostic bias or misdiagnoses may lead to spurious associations between conditions requiring clinical care and subsequent dementia diagnoses.

## Key Points

**Question:**

Is research on clinical factors associated with dementia incidence using electronic health records substantially biased by differential diagnosis or misdiagnosis?

**Findings:**

In this cohort study using data from the UK Biobank and All of Us involving 228 392 participants, diagnoses of conditions with varying risk associated with dementia were associated with increased short-term likelihood of incident dementia diagnosis. Most observed hazard ratios attenuated with time after exposure diagnosis, suggesting detection bias for dementia through health care utilization.

**Meaning:**

This cohort study offers empirical evidence of the plausible magnitude of detection bias for dementia based on 2 major cohorts increasingly widely used for dementia research.

## Teaser

This cohort study assesses whether detection bias or misdiagnosis bias occurs in the evaluation of factors associated with incident dementia when using electronic health record (EHR) data.

## Introduction

Electronic health record (EHR)–based studies on dementia have rapidly expanded.^1,2,3,4^ EHR data offer rich clinical information and a representation of patient health over time. However, EHR information systems engender distinct research challenges: clinical events are recorded only when patients have health care encounters, leading to potential detection bias.^5,6^

Detection bias—also called *informative presence bias* or *unmasking (detection signal) bias*—occurs when an exposure is associated with systematic differences in outcome ascertainment or diagnosis. For example, adults who would meet clinical criteria for dementia if they were assessed may not frequently interact with clinicians if they are otherwise healthy, thus delaying the dementia diagnosis. Diagnosed health conditions that bring patients into frequent interaction with health care may increase the chance that an individual receives a dementia diagnosis. This phenomenon can bias estimated associations of medical conditions or clinical care with subsequent dementia risk.^7,8,9,10^

Despite widespread acknowledgment of potential detection bias, there is a lack of evidence on the magnitude of this bias in EHR-based research on factors associated with dementia. Quantifying the impact of detection bias, or even plausible ranges, is essential for interpreting clinical research. Although it is methodologically challenging to evaluate the bias magnitude, we propose 2 strategies (eMethods in Supplement 1).

Our first approach contrasted EHR-derived estimated associations with estimates derived from existing cohort studies or meta-analyses of cohort studies that assessed dementia on a fixed schedule guided by the research design. Routinely scheduled dementia assessments should largely eliminate the type of detection bias in EHR-based research. Consequently, under detection bias, associations derived from EHR data may appear more positive than those from cohort studies. Misdiagnoses may create a similar phenomenon if clinical conditions, such as urinary tract infection (UTI), induce transient cognitive impairment that is misdiagnosed as dementia. We examined a spectrum of conditions with varying risk associated with dementia and their subsequent association with health care utilization. The conditions included well-established risk factors for dementia and factors with minimal plausible mechanisms linking to dementia in the spirit of negative control exposures.^11^

Our second approach to quantifying detection bias was premised on the expectation that detection bias will follow a distinctive time pattern, with the highest risk of new diagnoses immediately following clinical encounters and subsequently declining over time. For example, although diabetes may be associated with dementia risk, the effect is thought to accumulate over many years, with longer duration of diabetes associated with higher dementia risk.^12^ The association of health care encounters with dementia diagnosis, however, would likely peak around the time of diabetes diagnosis. The time pattern of outcome diagnoses vis-a-vis clinical encounters may thus offer insights into the magnitude of detection bias.

In this study, we aimed to estimate the plausible range of values of detection bias in EHRs by examining several clinical exposures: type 2 diabetes (T2D), depression, hypertension, UTI, kidney stones, forearm fracture, and gastrointestinal (GI) bleeding. We assessed associations of each exposure diagnosis with dementia using 2 EHR databases. In addition to assessing whether detection bias in EHR-based studies may affect research findings in dementia research, we also investigated whether each exposure was associated with health care utilization and whether health care utilization was associated with dementia diagnosis.

## Methods

The UK Biobank (UKB) study was approved by the North West Multi-Centre Research Ethics Committee, and all participants provided written informed consent. The All of Us (AOU) Research Program Institutional Review Board of the National Institutes of Health in the US approved all procedures, and all participants provided informed consent. This cohort study was based on deidentified data with no access to identifiers and therefore deemed not human participant research by the Boston University Institutional Review Board. This study followed the Strengthening the Reporting of Observational Studies in Epidemiology (STROBE) reporting guideline.

### Study Population and Participants

The UKB was a prospective cohort that enrolled over 500 000 participants aged 40 to 69 years from 2006 to 2010.^13^ At baseline, participants visited 1 of 22 UK assessment centers and completed questionnaires, interviews, and physical and medical assessments. Follow-up data included EHR-based hospital admission and primary care data and national death register data. Hospital admission data were available for all participants, while only around 45% of participants had primary care data linked to UKB. In our analysis, we included participants aged 55 years or older who were free of dementia at baseline and had both inpatient and primary care EHR data available. Participants were followed up from baseline (2006-2010) until December 2022.

The AOU Research Program is an ongoing large-scale research initiative enrolling diverse US participants starting from 2017, with data from baseline health surveys, physical measurements, and biospecimens and linkage to EHRs. Individuals aged 18 years or older residing in the US are eligible to enroll in the program online or at more than 340 recruitment sites.^14^ Participants completed baseline surveys at enrollment, and around 95% of them choose to provide authorization to share EHR data. In parallel with UKB data, our AOU analyses included participants aged 55 years or older who were free of dementia at baseline and had EHR data available. Participants were followed up from baseline (2017-2022) until July 2022. Additional data details are given in the eMethods in Supplement 1.

### Ascertainment of Exposures and Dementia Outcomes

For each dataset, we identified exposure and all-cause dementia diagnoses from participants’ EHRs. We identified the first documented incidence of each exposure diagnosis: T2D, depression, hypertension, UTI, kidney stones, forearm fracture, and GI bleeding. Dementia outcome included Alzheimer dementia, vascular dementia, Lewy body dementia, alcohol-related dementia, other types of dementia, and unspecified dementia. In the UKB, death records were also used to ascertain dementia diagnosis. Hospital inpatient and death records were coded using the *International Classification of Diseases, Ninth Revision* (*ICD-9*) and *International Statistical Classification of Diseases and Related Health Problems, Tenth Revision (ICD-10)* in UKB; primary care records were coded using Read, version 2 and Clinical Terms Version 3.^15^ In AOU, dementia and exposures were ascertained using *ICD-9*, *ICD-10*, and the corresponding Systematized Nomenclature of Medicine (SNOMED) codes. eTable 1 in Supplement 1 gives all source codes.

### Health Care Utilization

Because increased health care utilization was our primary hypothesized mechanism for detection bias, we evaluated whether the exposures were associated with the number of encounters with the health care system. In the UKB, we totaled the number of general practitioner and inpatient encounters. In the AOU, we counted all-cause patient encounters, including primary care records, inpatient records, procedures, and screenings. Participants with an incident exposure had their health care utilization calculated for 1 year prior to and 1 year after the exposure diagnosis. For those without a history of the exposure, health care utilization was calculated for 1 year prior to and 1 year after enrollment.

### Covariates

Our base covariate set included age, sex (female, male), number of apolipoprotein E epsilon 4 alleles (0, 1, or 2), and race and ethnicity. Race and ethnicity categories from both datasets are provided in eMethods in Supplement 1 and were collapsed for this analysis into Asian, Black, White, and other, which included mixed, other ethnic group, or any other mixed background in the UKB and Middle Eastern or North African, Native Hawaiian or Other Pacific Islander, more than 1 population, or none of these in the AOU. Race and ethnicity were included in the analysis because they are associated with both the exposures and dementia diagnosis. Our full models additionally adjusted for educational level (high school or above, less than high school), smoking history (ever smoked, never smoked), and continuous body mass index as a linear term. In the UKB, age and sex were acquired from the National Health Service Primary Care Trust registries and were updated by participants at baseline. AOU participants self-reported age and sex. Race and ethnicity (eMethods in Supplement 1), educational level, and smoking history were self-reported in both datasets using baseline surveys. Body mass index was constructed from height and weight measured during the baseline physical measurement in each dataset and was calculated as weight in kilograms divided by height in meters squared.

### Statistical Analysis

Analyses used R, version 4.4.0 (R Project for Statistical Computing) with the survival and dplyr packages. All statistical tests were 2-sided, with a significance level of α = .05. Data were analyzed from November 2023 through February 2025. In primary analyses, we adopted 2 approaches to assess detection bias: (1) comparing EHR-based estimated associations of clinical exposures with dementia diagnoses with previously published estimates from cohort studies when available and (2) evaluating how associations between incident clinical exposures and incident dementia diagnoses evolved over time. In a secondary analysis, we assessed associations of health care utilization with our exposures and incident dementia.

#### Primary Analysis

We summarized characteristics of each sample and calculated cumulative incidence of dementia while accounting for the competing risk of death. We used Cox proportional hazards regression models to examine associations between each exposure and incident dementia diagnosis. Participants were followed up from study baseline (date of enrollment into study) to the earliest of the date of the first dementia diagnosis, death from any cause, or administrative censoring, defined as the latest date of any dementia diagnosis in the sample (UKB, December 12, 2022; AOU, July 1, 2022). Each exposure diagnosis was time varying, with the exposure status updated if participants developed an incident diagnosis after enrollment (eMethods in Supplement 1).

We compared our EHR-derived estimated associations with benchmarks from existing meta-analyses of cohorts selected after a PubMed search (the eMethods in Supplement 1 give the search criteria). We used the most recent meta-analyses or the meta-analyses with the most included participants; if no meta-analysis was available, we used the most recently published cohort study. To investigate the pattern of detection bias over time for each exposure, we calculated hazard ratios (HRs) for incident dementia over 4 time intervals after the earliest exposure diagnosis (0-1 year, >1 to 5 years, >5 to 10 years, and >10 years after exposure diagnosis) compared with no prior exposure diagnosis.

In a preplanned analysis to establish a range of detection bias magnitudes in EHR data, we meta-analyzed the estimated associations of all exposures with dementia diagnoses from the full model using a random effects model with inverse variance weights. The estimated associations for the meta-analysis were calculated by dividing the HR from 0 to 1 year after the exposure diagnosis by the overall HR (estimated in a separate Cox proportional hazards regression model over the entire follow-up period) in our primary analysis, canceling the effects of potential residual confounding, true causal relationships, and other study biases commonly encountered in both estimates. Standard errors were calculated assuming independence.

#### Sensitivity Analyses for Primary Results

We conducted 4 sensitivity analyses. First, we hypothesized that individuals with high health care utilization would be less likely to have undiagnosed dementia and thus less vulnerable to detection bias when a new condition was diagnosed. Thus, we stratified our primary analysis by levels of health care utilization (low and high, by sample-specific median number of health care encounters during the year before baseline). Second, we restricted our analysis to participants with no prevalent exposure diagnosis at study baseline and evaluated the association of incident exposure with dementia diagnosis. Third, in our meta-analysis, instead of dividing the HR from 0 to 1 year after the exposure diagnosis by the overall HR, we replaced the overall HR with the HR from 1 to 2 years after the exposure and the HR derived from cohort studies or meta-analyses of cohort studies for established risk factors.^16,17,18,19^ Lastly, since the benchmark meta-analysis of T2D was stratified by sex, we stratified our primary analysis of T2D by sex.

#### Secondary Analyses

We conducted secondary analyses to investigate the associations of each exposure with health care utilization and the association of health care utilization with incident dementia. We used a difference-in-differences negative binomial regression model to compare the change in health care utilization among exposed groups with that in a control group.^20^ We compared the change in health care utilization before and after the incidence of each exposure in the exposed group with the change in health care utilization before and after baseline in the control group. Further details on the difference-in-differences design can be found in the eMethods in Supplement 1. To assess the association between health care utilization and dementia, we fit Cox proportional hazards regression models with the frequency of health care utilization 1 year prior to baseline as the exposure, adjusting for baseline age, sex, race and ethnicity, and educational level.

## Results

### Study Sample Characteristics

**Figure 1.**
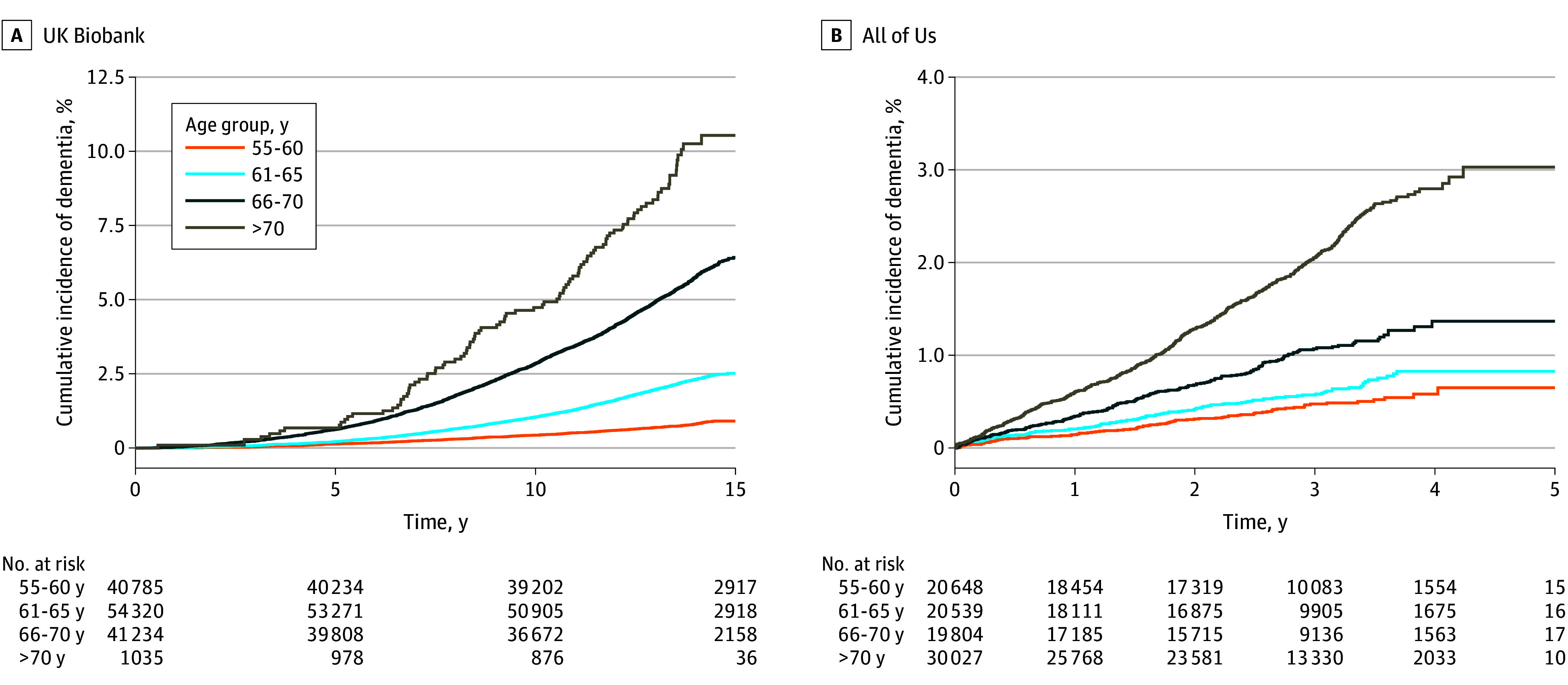
Cumulative Incidence of Dementia Over Time

In total, 137 374 UKB participants met the inclusion criteria. Mean (SD) age was 62.5 (4.1) years; 73 912 (53.8%) were female, and 63 462 (46.2%) were male (Table 1). The participants self-identified as Asian (2232 [1.6%]), Black (862 [0.6%]), White (133 053 [96.8%]), or other (1227 [0.9%]). The proportion of participants with a documented exposure diagnosis prior to baseline ranged from 1.6% for kidney stone (n = 2253) to 27.8% for hypertension (n = 38 218). Over a mean (SD) follow-up of 13.2 (2.2) years, 4095 new dementia diagnoses accrued, showing an age gradient (Figure 1).

**Table 1.** Characteristics of Participants in the Analytic Sample

Characteristic	Participants, No. (%)^a^	
	UK Biobank (n = 137 374)	All of Us (n = 91 018)
Age at baseline, mean (SD), y	62.5 (4.1)	66.9 (7.8)
Sex		
Female	73 912 (53.8)	51 946 (57.1)
Male	63 462 (46.2)	39 072 (42.9)
Race and ethnicity		
Asian	2232 (1.6)	1561 (1.7)
Black	862 (0.6)	15 733 (17.3)
White	133 053 (96.8)	61 853 (68.0)
Other^b^	1227 (0.9)	11 871 (13.0)
*APOE ε4* alleles, No.		
0	98 642 (71.8)	66 857 (73.4)
1	35 550 (25.9)	22 242 (24.4)
2	3182 (2.3)	1919 (2.1)
Educational level		
High school or more	81 210 (59.1)	83 372 (91.6)
<High school	56 164 (40.9)	7646 (8.4)
Smoking history		
Ever smoked	66 578 (48.5)	40 913 (45.0)
Never smoked	70 182 (51.1)	48 252 (53.0)
Missing	614 (0.4)	1853 (2.0)
BMI, mean (SD)	27.7 (4.7)	29.6 (6.8)
Follow-up time, mean (SD), y	13.2 (2.2)	2.7 (1.1)
Type 2 diabetes status		
No	119 713 (87.1)	69 189 (76.0)
Prevalent case before baseline	8455 (6.2)	18 811 (20.7)
Incident case during follow-up	9206 (6.7)	3018 (3.3)
Depression status		
No diagnosis	118 480 (86.2)	67 093 (73.7)
Prevalent case before baseline	11 511 (8.4)	20 495 (22.5)
Incident case during follow-up	7383 (5.4)	3430 (3.8)
Hypertension status		
No diagnosis	70 721 (51.5)	39 900 (43.8)
Prevalent case before baseline	38 218 (27.8)	45 563 (50.1)
Incident case during follow-up	28 435 (20.7)	5555 (6.1)
Urinary tract infection status		
No diagnosis	106 008 (77.2)	73 808 (81.1)
Prevalent case before baseline	16 276 (11.8)	14 146 (15.5)
Incident case during follow-up	15 090 (11.0)	3064 (3.4)
Kidney stone status		
No diagnosis	133 167 (96.9)	84 044 (92.3)
Prevalent case before baseline	2253 (1.6)	5498 (6.0)
Incident case during follow-up	1954 (1.4)	1476 (1.6)
Forearm fracture status		
No diagnosis	126 347 (92.0)	88 160 (96.9)
Prevalent case before baseline	6651 (4.8)	2180 (2.4)
Incident case during follow-up	4376 (3.2)	678 (0.7)
Gastrointestinal bleeding		
No diagnosis	128 281 (93.4)	82 095 (90.2)
Prevalent case before baseline	3201 (2.3)	7054 (7.8)
Incident case during follow-up	5892 (4.3)	1869 (2.0)
Encounters with the health care system, mean (SD), No.		
During the year before baseline	7.1 (7.7)	8.2 (14.7)
During the year after baseline	7.9 (8.2)	8.4 (14.6)

Abbreviations: *APOE ε4*, apolipoprotein E epsilon 4; BMI, body mass index (calculated as weight in kilograms divided by height in meters squared).

^a^ Percentages may not sum to 100 due to rounding.

^b^ The eMethods in Supplement 1 give additional information on race and ethnicity. In the UK Biobank, other included mixed, other ethnic group, and any other mixed background. In All of Us, other included Middle Eastern or North African, Native Hawaiian or Other Pacific Islander, more than 1 population, and none of these.

Among 91 018 AOU participants who met the inclusion criteria, the mean (SD) age was 66.9 (7.8) years; 51 946 (57.1%) were female, and 39 072 (42.9%) were male (Table 1). The participants self-identified as Asian (1561 [1.7%]), Black (15 733 [17.3%]), White (61 853 [68.0%]), and other (11 871 [13.0%]). The proportion of participants with a documented exposure diagnosis prior to baseline ranged from 2.4% for forearm fracture (n = 2180) to 50.1% for hypertension (n = 45 563). Over a mean (SD) follow-up of 2.7 (1.1) years, 952 new dementia diagnoses occurred (Figure 1). Among participants with a history of GI bleeding, the dementia incidence rates were 3.0 (UKB) and 7.7 (AOU) per 1000 person-years compared with 2.2 (UKB) and 2.4 (AOU) per 1000 person-years among those without a history of GI bleeding.

### Associations of Clinical Exposures With Dementia

**Figure 2.**
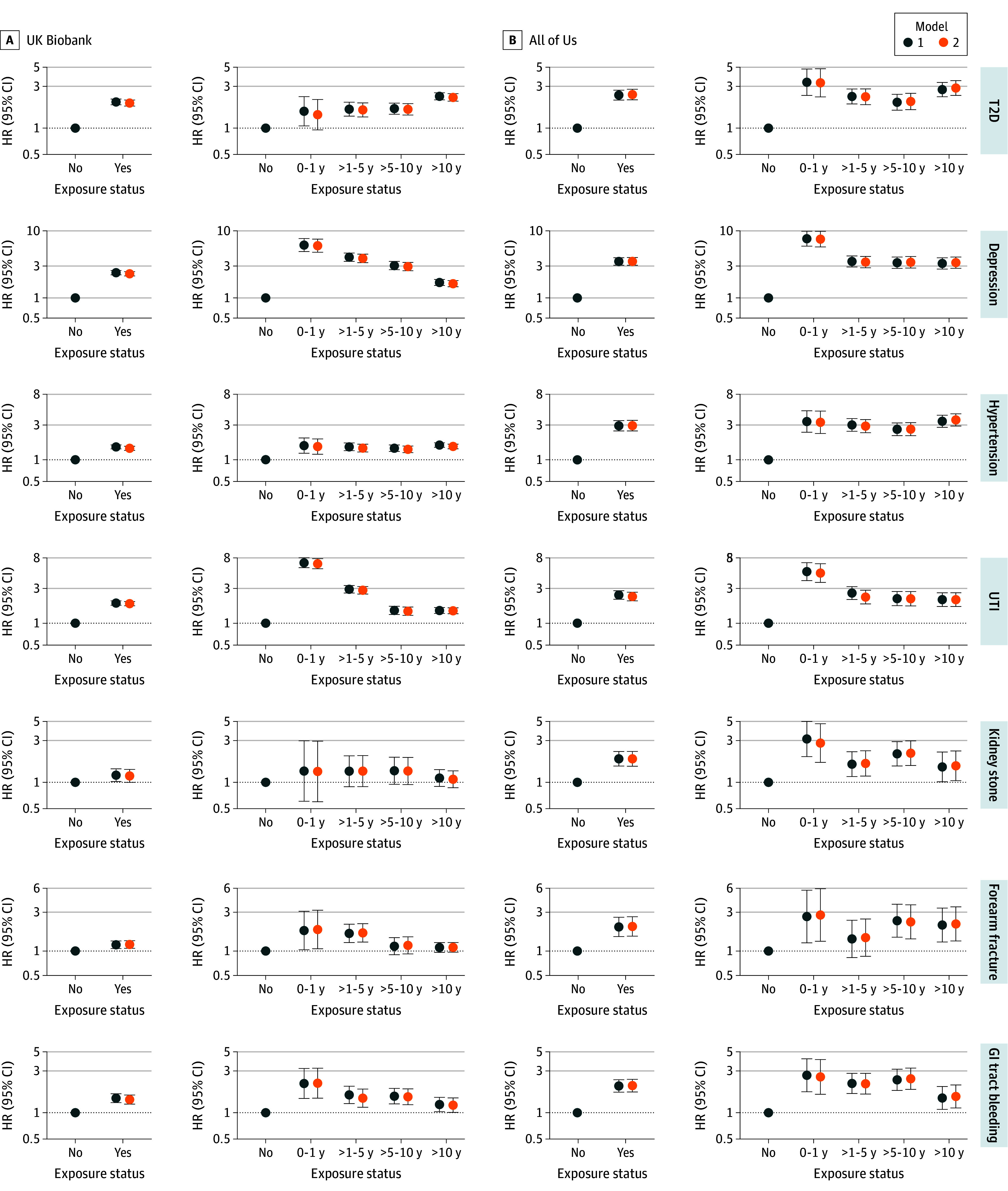
Associations of Clinical Exposures With Incidence of First Dementia Diagnosis Model 1 was adjusted for age, sex, race and ethnicity, and the number of apolipoprotein E epsilon 4 alleles. Model 2 was further adjusted for educational level, smoking history, and body mass index. The reference for overall and for time intervals was no exposure. GI indicates gastrointestinal; HR, hazard ratio; T2D, type 2 diabetes; and UTI, urinary tract infection.

In both datasets, all 7 clinical exposures showed a positive association with dementia incidence, with HRs ranging from 1.18 (95% CI, 1.00-1.40) to 3.51 (95% CI, 3.08-4.01). For instance, T2D was associated with a higher dementia hazard (HR, 1.94 [95% CI, 1.79-2.10] in UKB; HR, 2.43 [95% CI, 2.11-2.79] in AOU) (Figure 2 and eTable 2 in Supplement 1). The benchmark meta-analysis^16^ of 14 cohort studies showed that T2D was associated with a relative risk of 1.62 (95% CI, 1.45-1.80) for women and 1.58 (95% CI, 1.38-1.81) for men, both of which were lower than our EHR-derived estimates overall (eTable 3 in Supplement 1) and among male and female participants (eTable 5 in Supplement 1). In UKB, patients with at least 1 UTI episode had a greater hazard of dementia (HR, 1.85; 95% CI, 1.73-1.98) compared with participants without a UTI diagnosis; in AOU, this HR was 2.32 (95% CI, 2.02-2.67). Both EHR-based estimates were more pronounced than the estimated HR of 1.13 (95% CI, 1.08-1.18) from the benchmark cohort study.^19^

For most exposures, the highest incidence of dementia occurred in the first year following exposure diagnosis compared with no exposure diagnosis (Figure 2 and eTable 2 in Supplement 1). For example, participants diagnosed with GI bleeding had the highest dementia incidence in the first year after a GI bleeding diagnosis (HR, 2.17 [95% CI, 1.46-3.22] in UKB; HR, 2.56 [95% CI, 1.62-4.04] in AOU) (Figure 2 and eTable 2 in Supplement 1). Subsequently, during the 1 to 5 years following the diagnosis, the HR for the association of GI bleeding with incident dementia attenuated to 1.46 (95% CI, 1.15-1.86) in UKB and 2.14 (95% CI, 1.63-2.81) in AOU.

### Assessment of Detection Bias Magnitude From Meta-Analyses

**Figure 3.**
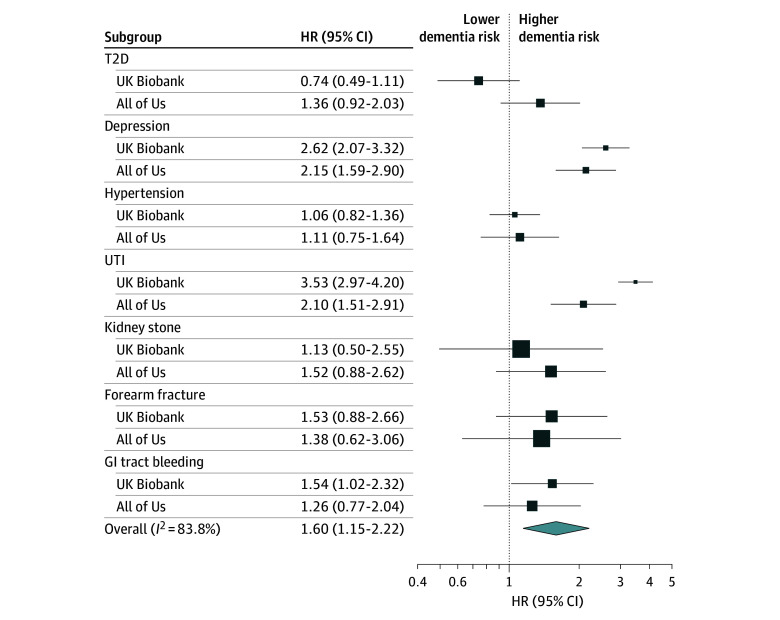
Forest Plot of Random Effects Models for the Pooled Detection Bias Estimates Point estimates for detection bias, which were used as inputs for the meta-analysis, were calculated by dividing the hazard ratio (HR) from 0 to 1 year after the exposure diagnosis by the overall HR, allowing for mitigation or cancellation of the effects of potential residual confounding, true causal relationships, and other study biases commonly encountered in both estimates. A random effects model with inverse variance weights was used. Size of squares represents the SE of the point estimate. GI indicates gastrointestinal; T2D, type 2 diabetes; and UTI, urinary tract infection.

The mean HR of each exposure estimate in the first year following the exposure to the long-term mean association when meta-analyzed across all exposures in both cohorts was 1.60 (95% CI, 1.15-2.22) (Figure 3 and eTable 4 in Supplement 1). In other words, in the first year after exposure diagnosis, the likelihood of receiving a dementia diagnosis was greater than the long-term average elevation in dementia risk associated with the exposure. However, HRs ranged from 0.74 (95% CI, 0.49-1.11) to 3.53 (95% CI, 2.97-4.20), and the *I^2^* statistic (ie, the percentage of variability in estimated associations due to heterogeneity in associations rather than sample size differences) was 83.8%.

### Sensitivity Analyses

Among participants with high levels of health care utilization before baseline, the HRs for clinical exposures associated with new dementia diagnoses were slightly lower than for overall associations (eFigure 1 in Supplement 1), resulting in a meta-analyzed estimated HR of 1.57 (95% CI, 1.07-2.30) (eFigure 2 in Supplement 1). Conversely, participants with low levels of health care utilization before baseline experienced greater estimated detection bias (meta-analyzed HR, 1.70 [95% CI, 1.31-2.20]) (eFigure 2 in Supplement 1). Excluding participants with prevalent exposure diagnosis at baseline did not substantially change the results (eFigure 3 in Supplement 1). Comparing dementia incidence at 0 to 1 year with 1 to 2 years after exposure (instead of the overall average estimate) led to a meta-analyzed estimated HR of detection bias of 1.41 (95% CI, 1.18-1.67) (eFigure 4 in Supplement 1), while comparing with existing meta-analyses for established risk factors^16,17,18,19^ increased the estimated HR to 2.02 (95% CI, 1.33-3.07) (eFigure 5 in Supplement 1).

### Secondary Analyses

The difference-in-differences estimates for changes in health care utilization between the incident exposure group and control group varied with exposure and health care utilization measurement. For most exposures, health care utilization increased after an incident exposure diagnosis increased compared with no exposure. For example, the frequency of health care utilization increased by 15.2% (95% CI, 11.0%-19.6%) (Table 2) in the year after forearm fracture diagnosis in UKB and by 27.7% (95% CI, 25.2%-30.2%) in AOU. Additionally, health care utilization during the year before baseline was associated with dementia incidence (UKB HR, 1.03 [95% CI, 1.02-1.03] per additional encounter; AOU HR, 1.02 [95% CI, 1.01-1.02] per additional encounter) (eTable 6 in Supplement 1).

**Table 2.** Comparison of Health Care Utilization Change 1 Year Before and 1 Year After Exposure Incidence

Exposure	DID estimate of change, % (95% CI)^a^	
	UK Biobank	All of Us
Type 2 diabetes	22.2 (19.0 to 25.5)	20.0 (18.8 to 21.2)
Depression	1.7 (−0.9 to 4.5)	13.3 (12.4 to 14.2)
Hypertension	10.0 (8.1 to 12.0)	15.0 (14.0 to 15.9)
Urinary tract infection	−2.1 (−4.2 to 0.0)	6.3 (5.3 to 7.3)
Kidney stone	0.9 (−4.4 to 6.5)	12.2 (10.8 to 13.7)
Forearm fracture	15.2 (11.0 to 19.6)	27.7 (25.2 to 30.2)
Gastrointestinal bleeding	−2.4 (−5.4 to 0.6)	17.0 (13.6 to 20.5)

Abbreviation: DID, difference in differences.

^a^ From negative binomial regression models adjusted for age, sex, race and ethnicity, and educational level.

## Discussion

We evaluated associations of 7 clinical exposures with incident dementia using 2 large EHR-based cohorts. All exposures, including those with minimal physiologic link to dementia, were positively associated with new dementia diagnoses recorded in the EHR. The estimated HRs were as large as those for many risk factors prioritized in a Lancet Commission report.^21^ The HRs for the observed associations were more pronounced compared with existing evidence from previously published cohort studies, and most observed HRs for the associations attenuated with time after exposure diagnosis.

Although associations observed for some exposures after longer follow-up may reflect, at least partially, the cumulative effects of the exposure, the short-term associations were more likely attributable to detection bias. Receipt of care for any condition brought the participant into contact with the health care system and, therefore, increased their likelihood of being diagnosed with dementia and created a spurious association between conditions requiring clinical attention and subsequent dementia diagnosis. Our results suggest that the magnitude of this detection bias may be substantial. We interpreted the meta-analyzed bias estimate centered at an HR of 1.60 (95% CI, 1.15-2.22) with great caution because the *I^2^* statistic of 83.8% suggested substantial heterogeneity in detection bias across exposures.^22^

Misdiagnoses may also have contributed to our results and would likewise introduce substantial bias in analyses of clinical factors associated with dementia incidence. In particular, transient cognitive dysfunction due to delirium following acute conditions, such as UTIs and fractures^23,24^; cognitive impairment due to hemorrhage following GI bleeding^25^; and cognitive symptoms associated with depression^26^ could contribute to the observed short-term increase in dementia diagnoses.

While the existence of detection bias has been well documented in prior literature,^7,8,27,28,29,30,31^ few studies have offered a plausible range of its magnitude or derived estimates from clinical data. Such an approach is challenging because it is impossible to conclusively disentangle bias from true associations in clinical data. For instance, hypertension is a known risk factor for dementia.^21^ Individuals with a history of hypertension may exhibit higher dementia diagnosis rates due to an increased risk, because greater health care utilization increases their likelihood of receiving a diagnosis, or both. However, if dementia is diagnosed shortly after hypertension diagnosis, the timing suggests that detection bias, including earlier detection, is part of the reason. Our approach acknowledges that no single association provides conclusive information and that the magnitude of the bias varies by condition. The approach posits health care utilization as a common mechanism for the bias and uses unexpected associations across multiple exposures to provide insight into its magnitude.

Dementia has a long preclinical phase, and overlapping and evolving symptoms and social factors may delay diagnosis.^32,33^ Individuals exhibiting clinical symptoms closer to the diagnostic threshold who are concerned about their cognitive decline or dementia or whose caregivers express concerns are more likely to receive a dementia diagnosis.^33,34^ This dynamic creates a contact-dependent nature to dementia diagnosis. Increased likelihood of diagnosis may arise from more opportunities to observe dementia symptoms; from the metabolic, emotional, or cognitive stress associated with illness or treatments; or from referrals to specialists, particularly for conditions with overlapping clinical symptoms.

Our findings demonstrate the potential value of periodic direct cognitive assessments embedded in EHR-based follow-up studies. To assess potential bias from misdiagnosis of transient cognitive symptoms, it may be helpful to require multiple diagnoses across a minimum period. Future research leveraging more detailed clinical data may offer more specific quantification of the magnitude of such bias across additional risk factors and samples. Furthermore, future studies should assess the extent to which detection bias is acting exclusively through increased health care utilization. Studies using EHR data for epidemiologic and clinical research should exercise caution and thoughtful consideration regarding context and potential limitations.

### Limitations

Key limitations of this study include possible residual confounding, lack of evaluation of differences across health care settings, possible missing EHR records, and use of convenience samples in both cohorts. We cannot rule out the presence of other study biases, such as residual confounding by frailty.^35^ Our analyses did not differentiate between health care encounters across different types of facilities (eg, inpatient, outpatient, or emergency department), which could influence the probability of receiving a dementia diagnosis.^36,37^ This limitation restricted our ability to explore the detailed mechanisms underlying the bias. EHR data may be incomplete. Also, a potential healthy volunteer selection bias was possible.^38,39^ None of these limitations, however, could plausibly explain our primary results.

## Conclusions

In this cohort study of 2 large datasets, diagnoses of several conditions associated with varying risks of dementia were associated with a higher short-term likelihood of dementia diagnosis. The findings suggest a potential detection bias in analyses of EHRs when evaluating clinical factors associated with dementia. Detection bias should be directly evaluated and addressed in EHR-based research on clinical risk factors. Our strategies can be adapted to detect such bias, and analytical approaches, such as estimating time-varying exposures, may mitigate it.

## Notes

Supplement 1.
eMethods.
**eTable 1.** ICD, Read v2, CTV3, and SNOMED codes for outcome and exposures **eTable 2.** Associations of exposures with dementia incidence **eTable 3.** Benchmark estimates derived from existing meta-analyses of cohort studies **eTable 4.** Pooled detection bias estimates in random-effects models **eTable 5.** Association between type 2 diabetes mellitus and incident dementia, stratified by sex **eTable 6.** Association between health care utilization one year prior to baseline and incident dementia **eFigure 1.** Associations of exposures with dementia incidence, stratified by levels of health care utilization **eFigure 2.** Forest plot of random-effects models for the pooled detection bias estimates, stratified by levels of health care utilization **eFigure 3.** Associations of exposures with dementia incidence, excluding prevalent exposure cases **eFigure 4.** Forest plot of random-effects models for the pooled detection bias estimates, comparing with estimates 1-2 year after exposure diagnosis **eFigure 5.** Forest plot of random-effects models for the pooled detection bias estimates, comparing with estimates from meta-analysis of cohort studies

Supplement 2.
Data Sharing Statement
