# Supplementary material for: Detection Bias in EHR-Based Research on Clinical Exposures and Dementia
Source: JAMA Netw Open. 2025 Apr 23;8(4):e256637. doi: 10.1001/jamanetworkopen.2025.6637 (PMC12019524; doi:10.1001/jamanetworkopen.2025.6637)
Supplement: Supplement 1. — eMethods. eTable 1. ICD, Read v2, CTV3, and SNOMED codes for outcome and exposures eTable 2. Associations of exposures with dementia incidence eTable 3. Benchmark estimates derived from existing meta-analyses of cohort studies eTable 4. Pooled detection bias estimates in random-effects models eTable 5. Association between type 2 diabetes mellitus and incident dementia, stratified by sex eTable 6. Association between health care utilization one year prior to baseline and incident dementia eFigure 1. Associations of exposures with dementia incidence, stratified by levels of health care utilization eFigure 2. Forest plot of random-effects models for the pooled detection bias estimates, stratified by levels of health care utilization eFigure 3. Associations of exposures with dementia incidence, excluding prevalent exposure cases eFigure 4. Forest plot of random-effects models for the pooled detection bias estimates, comparing with estimates 1-2 year after exposure diagnosis eFigure 5. Forest plot of random-effects models for the pooled detection bias estimates, comparing with estimates from meta-analysis of cohort studies [file jamanetwopen-e256637-s001.pdf]

Supplementary Online Content

Wang J, Choi M, Buto P, et al. Detection bias in EHR-based research on clinical exposures and dementia. *JAMA Netw Open*. 2025;8(4):e256637. doi:10.1001/jamanetworkopen.2025.6637

eMethods.
eTable 1. ICD, Read v2, CTV3, and SNOMED codes for outcome and exposures
eTable 2. Associations of exposures with dementia incidence
eTable 3. Benchmark estimates derived from existing meta-analyses of cohort studies
eTable 4. Pooled detection bias estimates in random-effects models
eTable 5. Association between type 2 diabetes mellitus and incident dementia, stratified by sex
eTable 6. Association between healthcare utilization one year prior to baseline and incident dementia
eFigure 1. Associations of exposures with dementia incidence, stratified by levels of healthcare utilization
eFigure 2. Forest plot of random-effects models for the pooled detection bias estimates, stratified by levels of healthcare utilization
eFigure 3. Associations of exposures with dementia incidence, excluding prevalent exposure cases
eFigure 4. Forest plot of random-effects models for the pooled detection bias estimates, comparing with estimates 1-2 year after exposure diagnosis
eFigure 5. Forest plot of random-effects models for the pooled detection bias estimates, comparing with estimates from meta-analysis of cohort studies
eReferences.

This supplementary material has been provided by the authors to give readers additional information about their work.

© 2025 Wang J et al. *JAMA Network Open*.

eMethods

1. Two strategies to offer insight into the magnitude of detection bias

**Strategy**	**Description**	**Hypothesized results under detection bias**	**Hypothesized results under no detection bias**
Estimated associations comparison	Contrasting EHR-derived effect estimates to those derived from previously published cohort studies or meta-analyses of cohort studies, which are less susceptible to detection bias.	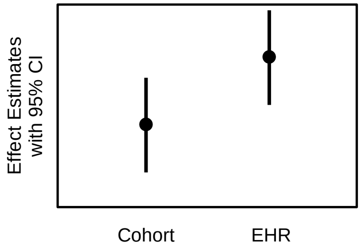	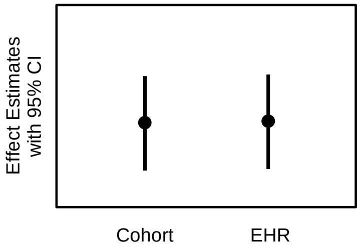
Time pattern investigation	Evaluating the temporal pattern of exposure-outcome associations, which we expect to show the largest impact on new diagnoses immediately following clinical encounters, with subsequent decline over time.	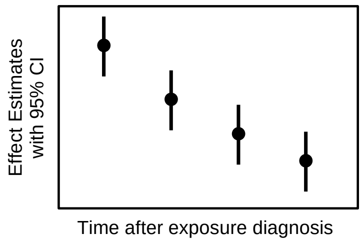	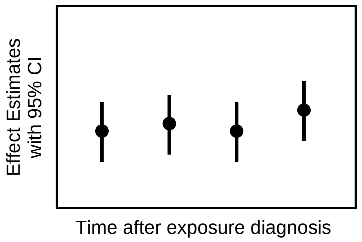

© 2025 Wang J et al. *JAMA Network Open*.

2. UK Biobank and All of Us data

UK Biobank

In the UK, all residents have the right to free NHS primary care services, with 98% of the population registered with a general practitioner (GP). GPs are typically responsible for the initial assessment, diagnosis, and treatment across a broad spectrum of medical conditions. Although there is no unified system for collecting or sharing primary care data, the UK Biobank has managed to obtain primary care data for around 45% of its participants. To compare participants with and without primary care data linkage, we summarized the baseline characteristics of all participants who were 55 years or older and free of dementia at baseline, stratified by linkage status. All demographic characteristics were similar among participants with and without linkage. For most exposures, the proportion of prevalent exposure diagnosis among participants without primary care data linkage was smaller than those among participants with primary care data linkage.

Characteristic *n (%)*[a]	UK Biobank participants who were 55 years or older and free of dementia at baseline and **with** primary care data	UK Biobank participants who were 55 years or older and free of dementia at baseline and **without** primary care data
N	137374	161410
Mean age at baseline (SD)	62.5 (4.08)	62.6 (4.09)
Sex		
Female	73912 (53.8%)	85989 (53.3%)
Male	63462 (46.2%)	75421 (46.7%)
Race		
Asian	2232 (1.6%)	2817 (1.7%)
Black	862 (0.6%)	2046 (1.3%)
Other	1227 (0.9%)	1991 (1.2%)
White	133053 (96.9%)	154556 (95.8%)
APOE-ε4 alleles		
0	98642 (71.8%)	115836 (71.8%)
1	35550 (25.9%)	41763 (25.9%)
2	3182 (2.3%)	3811 (2.4%)

© 2025 Wang J et al. *JAMA Network Open*.

Education		
High school or more	81210 (59.1%)	97035 (60.1%)
Less than high school	56164 (40.9%)	64375 (39.9%)
Smoking history		
Ever smoked	66578 (48.5%)	78265 (48.5%)
Never smoked	70182 (51.1%)	82338 (51.0%)
Missing	614 (0.4%)	807 (0.5%)
Mean BMI (SD)	27.7 (4.67)	27.5 (4.63)
Mean follow-up time (SD)	13.2 (2.24)	13.1 (2.40)
Type 2 diabetes mellitus status		
No	119713 (87.1%)	143390 (88.8%)
Prevalent case before baseline	8455 (6.2%)	5119 (3.2%)
Incident case during follow-up	9206 (6.7%)	12901 (8.0%)
Depression status		
No	118480 (86.2%)	151251 (93.7%)
Prevalent case before baseline	11511 (8.4%)	1350 (0.8%)
Incident case during follow-up	7383 (5.4%)	8809 (5.5%)
Hypertension status		
No	70721 (51.5%)	92844 (57.5%)
Prevalent case before baseline	38218 (27.8%)	17632 (10.9%)
Incident case during follow-up	28435 (20.7%)	50934 (31.6%)
Urinary tract infection status		
No	106008 (77.2%)	148338 (91.9%)
Prevalent case before baseline	16276 (11.8%)	2593 (1.6%)
Incident case during follow-up	15090 (11.0%)	10479 (6.5%)
Kidney stone status		
No	133167 (96.9%)	158107 (98.0%)

© 2025 Wang J et al. *JAMA Network Open*.

Prevalent case before baseline	2253 (1.6%)	1251 (0.8%)
Incident case during follow-up	1954 (1.4%)	2052 (1.3%)
Forearm fracture status		
No	126347 (92.0%)	157151 (97.4%)
Prevalent case before baseline	6651 (4.8%)	1378 (0.9%)
Incident case during follow-up	4376 (3.2%)	2881 (1.8%)
Gastrointestinal bleeding		
No	128,281 (93.4%)	155,476 (96.3%)
Prevalent case before baseline	3,201 (2.3%)	369 (0.2%)
Incident case during follow-up	5,892 (4.3%)	5,565 (3.4%)
Number of encounters with the healthcare system during the year before baseline (SD)	8.40 (8.89)	0.29 (1.54)

All of Us (AOU)
Approximately 95.8% AOU participants consented to share their EHR data, allowing for health outcomes to be identified through EHRs. All AOU EHR data were harmonized across sites using the Observational Medical Outcomes Partnership (OMOP) Common Data Model.[1]

© 2025 Wang J et al. *JAMA Network Open*.

3. **Racial and ethnic data details and reporting**

In both datasets, self-reported race and ethnicity categories were collapsed into the following categories: Asian, Black, White, and Other. The detailed categories and categorization are shown in the table below.

Categorization	UK Biobank	All of Us
Asian	Asian or Asian British, Chinese, Indian, Pakistani, Bangladeshi, White and Asian, Any other Asian background	Asian
Black	Black or Black British, Caribbean, African, White and Black Caribbean, White and Black African, Any other Black background	Black or African American
White	White, British, Irish, Any other white background	White
Other	Mixed, Other ethnic group, Any other mixed background	Middle Eastern or North African, More than one population, Native Hawaiian or Other Pacific Islander, None of these

© 2025 Wang J et al. *JAMA Network Open*.

4. Time-varying exposure

Each exposure diagnosis was time-varying, with the exposure status updated if participants developed an incident diagnosis after enrollment: those with an incident exposure diagnosis contributed person-time to the “unexposed” group until their date of exposure diagnosis, after which they contributed to person-time in the incident exposure group. Participants with prevalent exposure, i.e., who had a documented exposure diagnosis before baseline, were included in the primary analyses.

© 2025 Wang J et al. *JAMA Network Open*.

5. Search criteria for benchmark meta-analyses

Eligibility for the benchmark comparison estimates included: meta-analysis or cohort study if no meta-analysis exists, all-cause dementia as the outcome, and each clinical condition (type 2 diabetes mellitus, depression, hypertension, urinary tract infection, kidney stones, and forearm fracture) as the exposure.

Search criteria was defined as follows:

((meta-analysis) AND (dementia[MeSH Terms]) AND (type 2 diabetes[MeSH Terms]))

((meta-analysis) AND (dementia[MeSH Terms]) AND (depression[MeSH Terms]))

((meta-analysis) AND (dementia[MeSH Terms]) AND (hypertension[MeSH Terms]))

((meta-analysis) AND (dementia[MeSH Terms]) AND (urinary tract infection [MeSH Terms]))

((meta-analysis) AND (dementia[MeSH Terms]) AND (kidney stone[MeSH Terms]))

((meta-analysis) AND (dementia[MeSH Terms]) AND (forearm fracture[MeSH Terms]))

((meta-analysis) AND (dementia[MeSH Terms]) AND (gastrointestinal bleeding[MeSH Terms]))

© 2025 Wang J et al. *JAMA Network Open*.

6. **Difference-in-difference (DID) design for the associations between exposure diagnosis and healthcare utilization**

Due to the count data nature for the number of encounters with the healthcare system, the association of each exposure on healthcare utilization was estimated from an adjusted DID negative binomial regression model:

$$\ln(Y_{it}) = \beta_0 + \beta_1 \times X_i + \beta_2 \times D_t + \beta_3 \times X_i \times D_t$$

$$+ \beta_4 \times age_i + \beta_5 \times D_t \times age_i + \beta_6 \times sex_i + \beta_7 \times race_i + \beta_8 \times educ_i + \epsilon_{it},$$

where Y_{it} is the measurement of healthcare utilization of participant i at time t. X_i is a binary indicator (1=exposed group, 0=control group), D_t is a binary dummy variable (1=after incidence or baseline, 0=before incidence or baseline), age_i , sex_i, $race_i$, and $educ_i$ are individual baseline age, sex, race, and education, respectively. The DID estimate for the association between exposure diagnosis and healthcare utilization is represented by the parameter β_3.

© 2025 Wang J et al. *JAMA Network Open*.

eTable 1. ICD, Read v2, CTV3, and SNOMED codes for outcome and exposures

	ICD-9	ICD-10	Read v2	CTV3	SNOMED
**Outcome**					
Dementia	331.0, 290.4, 331.1, 046.1, 291.1, 291.2, 290.0, 290.1, 290.2, 290.3, 290.8, 290.9, 294.1, 294.8, 331.2	F00, F00.0, F00.1, F00.2, F00.9, G30, G30.0, G30.1, G30.8, G30.9, F01, F01.0, F01.1, F01.2, F01.3, F01.8, F01.9, I67.3, F02.0, G31.0, A81.0, F02.1, F02.2, F02.3, F02.4, F10.6, F02, F02.8, F03, F05.1, G31.1, G31.8	F110.,F1100,F1101,Eu00.,Eu000,Eu001,Eu002,Eu00z,Fyu30,E004.,E0040,E0041,E0042,E0043,E004z,Eu01.,Eu010,Eu011,Eu012,Eu013,Eu01y,Eu01z,F21y2,G678.,Eu020,F111.,A411.,F11x7,E011.,E0110,E0111,E0112,E011z,E012.,E0120,Eu021,A4110,Eu022,Eu023,Eu024,F11x9,Eu106,E000.,E001.,E0010,E0011,E0012,E0013,E001z,E002.,E0020,E0021,E002z,E003.,E00y.,E00z.,E041.,E04y.,Eu02.,Eu025,Eu02y,F116.,Eu02z,Eu041,F112.,F10y.,F10y0,F10y1,F10y2,F10yz,F118.,F11y.,F11y2,F11yz,Fyu31,E00..,1461.,38C13,3AE3.,3AE4.,3AE5.,3AE6.,66h..,6AB..,8BM02,8BM50,8BM60,8BPa.,8CMe0,8CMG2,8CMZ.,8CMZ0,8CMZ1,8CMZ2,8CMZ3,8CSA.,8HIa.,8IAe0,8IAe2,9hD..,9hD0.,9hD1.,9Ou..,9Ou1.,9Ou2.,9Ou3.,9Ou4.,9Ou5.	F110.,XaIKB,XaIKC,Eu00.,X002x,X002x,X002x,X002x,X002x,X002x,X002x,X0030,X0030,X0030,X0030,X0030,Eu00.,X0030,X0030,Eu002,Eu00z,Eu00z,Fyu30,XE1Xs,Xa0lH,E0040,E0041,E0042,E0043,E004z,XE1Xs,XE1Xs,XE1Xs,X003R,Xa0lH,Xa0lH,Xa0lH,X003T,X003V,Eu01y,Eu01z,F21y2,F21y2,XaIRJ,Eu020,F111.,A411.,F11x7,E011.,E011.,E011.,E011.,E0111,E0112,E011z,XE1Xu,X00Rk,E0120,Eu021,XabVp,XabVp,Eu022,Eu023,X003P,X003P,XaOfZ,Eu106,Eu106,Eu106,E000.,E001.,E0010,E0011,E0012,E0013,E001z,E002.,E0020,E0021,E002z,E003.,XE1Xt,X00R0,E00z.,E041.,E04y.,Eu02.,XaKyY,Eu02y,X003A,XE1Z6,XE1Z6,E00z.,E00z.,Eu02z,Xa1GB,Eu02z,X00R2,XE1Z6,E00z.,Eu02z,XE1Xr,Eu041,F112.,F10y.,F10y0,F10y1,XaPws,F10yz,X0037,F11..,X003m,F11yz,Fyu31,XE1Xr,X00R2,X002w,1461.,XaaeA,XaJBU,XaJBV,XaJBW,XaJBX,XaMJC,XaMGF,XabtQ,Xaefu,Xaefv,XaaiW,XacLx,XabEl,XaaBZ,XacIx,XacIy,XacIz,XacJ0,XabEk,XaYFR,XabEi,XacM2,XaLFf,XaLFo,XaLFp,XaMFy,XaMG0,XaMGG,XaMGI,XaMGJ,XaMGK	4182210

© 2025 Wang J et al. *JAMA Network Open*.

Exposure					
T2DM	250.x0, 250.x2	E11	C1001,C1011,C1021,C1031,C1041,C1051,C1061,C1071,C1072,C1074,C109.,C1090,C1091,C1092,C1093,C1094,C1095,C1096,C1097,C1099,C109A,C109B,C109C,C109D,C109E,C109F,C109G,C109H,C109J,C109K,C10F.,C10F0,C10F1,C10F2,C10F3,C10F4,C10F5,C10F6,C10F7,C10F8,C10F9,C10FA,C10FB,C10FC,C10FD,C10FE,C10FF,C10FG,C10FH,C10FJ,C10FK,C10FL,C10FM,C10FN,C10FP,C10FQ,C10FR,C10FS,C10P1,C10Q.,C10y1,C10z1,C1...,C10..,C100.,C1000,C1001,C100z,C101.,C1010,C1011,C101y,C101z,C102.,C1020,C1021,C102z,C103.,C1030,C1031,C103y,C103z,C104.,C1040,C1041,C104y,C104z,C105.,C1050,C1051,C105y,C105z,C106.,C1060,C1061,C106y,C106z,C107.,C1070,C1071,C1072,C1073,C1074,C107y,C107z,C108.,C1080,C1081,C1082,C1083,C1084,C1085,C1086,C1087,C1088,C1089,C108y,C108z,C109.,C1090,C1091,C1092,C1093,C1094,C1095,C1096,	66AJ2,C10..,C100.,C1001,C101.,C1011,C102.,C1021,C103.,C1031,C1041,C1041,C105.,C1051,C1051,C1061,C1071,C1072,C1074,C1090,C1090,C1091,C1092,C1093,C1094,C1095,C1096,C1096,C1097,C1097,C10y1,C10y1,C10y1,C10z.,C10z1,F1711,F1711,F3450,F3450,F3720,F3720,F3721,F3721,F3722,F3722,F420.,F420.,F4200,F4200,F4201,F4201,F4202,F4202,F4203,F4203,F4407,F4407,F4640,F4640,G73y0,G73y0,K01x1,K01x1,M0372,M2710,M2711,M2712,N0300,N0300,N0301,N0301,R0542,R0543,X00Ag,X00Ag,X00Ah,X00Ah,X00Ai,X00Ai,X00Aj,X00Aj,X00Ak,X00Ak,X00Al,X00Al,X00Am,X00Am,X00An,X00An,X00dF,X00dF,X00dG,X00dG,X00dH,X00dH,X00dI,X00dI,X00dJ,X00dJ,X30Kk,X30Kk,X30Kl,X30Kl,X30Km,X30Km,X40J5,X40J5,X40J6,X40J6,X40JJ,X40JJ,X40JQ,X40JR,X40JV,X40Ja,X40Jb,X40Jq,X40Jr,X40Js,X40KG,X5086,X5086,X50GO,X50GP,X50GP,X50GQ,X50GQ,X50GR,X50GR,X50GT,X50GT,XE10F,XE10G,XE10G,XE10H,XE10I,XE12M,XE15k,XE15k,XE15n,XE15n,XE1T3,XM0q4,XM1Qx,XSETH,XSETH,Xa0lK,Xa0lK,Xa1J5,Xa3ee,XaBLf,XaBLg,XaBuI,XaBuI,XaCJ2,XaD2T,XaD2T,XaE5T,XaE5T,XaE5U,XaE5U,XaE5V,XaE5V,XaE5W,XaE5W,XaE5X,XaE5X,XaE5Y,XaE5Y,XaE5Z,XaE5a,XaE5a,XaE5c,XaE5c,XaELQ,XaEVO,XaEVO,XaEVP,XaEVP,XaEVS,XaEVS,XaEVT,XaEVT,XaEnp,XaEnp,XaEnq,XaEnq,XaF05,XaF05,XaF97,XaF97,XaFWI,XaFmA,XaFmA,XaFn7,XaFn7,XaFn8,XaFn8,XaFn9,XaFn9,XaI	201826

© 2025 Wang J et al. *JAMA Network Open*.

			C1097,C10A.,C10A0,C10A1,C10A2,C10A3,C10A4,C10A5,C10A6,C10A7,C10AW,C10AX,C10y.,C10y0,C10y1,C10yy,C10yz,C10z.,C10z0,C10z1,C10zy,C10zz,F3y0.,G73y0,R0542,R0543	P5,XaIP5,XaIPk,XaIPk,XaIW8,XaIW8,XaIeJ,XaIeK,XaIrf,XaIyz,XaIyz,XaIz0,XaIz0,XaIzQ,XaIzQ,XaIzR,XaIzR,XaJOg,XaJOg,XaJOh,XaJOh,XaJOi,XaJOi,XaJOj,XaJOj,XaJOk,XaJOk,XaJOl,XaJOl,XaJOn,XaJOn,XaJOo,XaJOo,XaJQp,XaJQp,XaKDG,XaKDG,XaKDH,XaKDH,XaKDI,XaKDI,XaKDJ,XaKDJ,XaKHh,XaKHi,XaKcS,XaKcS,XaKyX,XaOPt,XaPen,XaPen,XaPmW,XaPmW,XaPmX,XaPmX,XaXbW,XaXbW,XaXfs,XaXfs,Xaa8r,XaaEs,XaaEt,XaaEu,XaaEv,XaaEw,Xaagf,Xaagf,Xac0x,Xac0x,XacoB,XacoB,66AJ1,66AJ2,C10..,C10..,C100.,C100z,C101.,C1010,C1011,C101y,C101z,C102.,C1020,C1021,C102z,C103.,C1030,C1031,C103y,C103z,C1040,C1041,C104y,C104z,C105.,C1050,C1051,C105y,C105z,C1060,C1061,C106y,C106z,C1070,C1071,C1072,C1073,C1074,C107y,C107z,C1080,C1081,C1082,C1083,C1085,C1086,C1087,C1088,C108y,C108z,C1090,C1091,C1092,C1093,C1094,C1095,C1096,C1097,C10A0,C10A1,C10A2,C10A3,C10A4,C10A5,C10A6,C10A7,C10y.,C10y0,C10y1,C10yy,C10yz,C10z.,C10z0,C10z1,C10zy,C10zz,C362.,C3622,C3626,Cyu21,Cyu22,Cyu23,F1711,F3450,F35z0,F3720,F3721,F3722,F420.,F4200,F4201,F4202,F4203,F420z,F4407,F4640,G73y0,K01x1,L1805,L1806,L1807,L1808,Lyu29,M0372,M2710,M2711,M2712,N0300,N0301,R0542,R0543,X00Af,X00Ag,X00Ah,X00Ai,X00Aj,X00Ak,X00Al,X00Am,X00An,X00FH,X00dF,X00dG,X00dH,X00dI,X00dJ,X203R,X203S,X203T,X203U,X30Kk,X30Kl,X30Km,X40J4,X40J4,X40J5,X40J5,X40J6,X40J6,X40J7,X40J7,X40J8,X40J9,X40J9,X40JE,X40	

© 2025 Wang J et al. *JAMA Network Open*.

				JI,X40JI,X40JJ,X40JJ,X40JN,X40JO,X40JP,X40JQ,X40JT,X40JV,X40JW,X40JX,X40JY,X40Jb,X40Jc,X40Jc,X40Jj,X40Jj,X40Jq,X40Jr,X40PF,X40PS,X40PT,X40PU,X40PW,X5085,X5086,X5087,X50GO,X50GP,X50GQ,X50GR,X50GT,X702N,X702P,X789v,XE10E,XE10F,XE10G,XE10H,XE10I,XE15k,XE15n,XM07q,Xa0lK,Xa1J5,Xa1J5	
Depression	296.2, 296.3	F32, F33	Eu32.,Eu320,Eu321,Eu322,Eu323,Eu324,Eu325,Eu326,Eu327,Eu328,Eu329,Eu32B,Eu32y,Eu32z,E114.,E1140,E1141,E1142,E1143,E1144,E1145,E1146,E114z	2257.,2257.,E0043,E0043,E1121,E1122,E1123,E1124,E1126,E11y2,E130.,E2B0.,E2B1.,Eu320,Eu321,Eu322,Eu323,Eu32y,Eu32z,X00Qy,X00SO,X00SO,X00SQ,X00SU,XE1Y0,XE1YC,XE1YC,XE1ZY,XE1ZZ,XE1Za,XE1Zb,XSEGJ,XSGok,XSGol,XSGom,XSGon,XaB9J,XaB9J,XaCHr,XaCHs,XaCIs,XaCIt,XaCIt,XaCIu,XaCIu,XaX53,XaY2C,E114.,E1140,E1141,E1142,E1143,E1144,E1145,E1146,E114z,E2113,Eu310,Eu311,Eu312,X00SM,X00SN	440383, 3656234, 4269493, 4148630, 4176002, 4323418, 37111697, 4282096, 4152280, 4149320, 4336957, 4195572, 4228802, 4338031, 4151170, 4307111, 4049623, 4077577, 4098302, 4282316, 4263748, 4141454, 433991, 4149321, 42872722, 4250023, 4327337, 438406, 441534, 43531624, 435220, 4025677
Hypertension	401.x-405.x	I10, I158, I159	G24..,G240.,G240z,G241.,G241z,G24z.,G24z1,G24zz,Gyu20,G2...,G20..,G200.,G201.,G202.,G203.,G20z.,G25..,G250.,G251.,G26..,G27..,G28..,G2y..,G2z..,Gyu2.,G2...,G20..,G200.,G201.,G202.,G20z.,G24..,G240.,G2400,G240z,G241.,G2410,G241z,G244.,G24z.,G	61462,G24..,G240.,G240z,G241.,G241z,G24z.,G24z1,Gyu20,G2...,G20..,G200.,G201.,G202.,G20z.,G2y..,G2z..,G5802,G5803,X50Bh,XE0Ub,XE0Uc,XE0Ud,XSDSb,Xa0Cs,Xa3fQ,XaIWn,XaZWm,XaZWn,XaZbT,XaZbz,XaZzo,Xab9L,Xab9M,G200.,G201.,G24..,G24..,G240.,G2400,G240z,G241.,G241z,G244.,G24z.,G24z0,G2y..,G2y..,G2y..,G2z..,G2z..,G2z..,Gyu21,X206H,X206H,X206H,X206I,X206I,X206J,X206J,X206K,X206K	312648, 4028741, 320128, 316866, 319826

© 2025 Wang J et al. *JAMA Network Open*.

			24z0,G24z1,G24zz,G2y..,G2z..	,X50Bh,X50Bh,X50Bh,XE0Ub,XE0Ub,XE0Uc,XE0Uc,XE0Ud,Xa0Cs,Xa0Cs,Xa0Cs	
UTI	595.0, 595.9, 599.0	N30.0, N30.9, N39.0	K15..,K150.,K155.,K15z.,K190.,K1900,K1901,K1902,K1903,K1904,K1905,K1906,K190z,K1z..,SP07Q,K150.,K155.,K15z.,K190.,K1900,K1901,K1903,K1904,K190z	K15..,K15z.,K190.,K1900,K1901,K1902,K1903,K1904,K190z,X30NV,X30NY,X30NZ,X30Na,X30PX,X30PZ,X30Pa,X30Pe,XE0e0,XE0e1,XM0sZ,XM0sc,XM1VF,Xa7nb,Xa7nc,Xa7nd,Xa7ne,Xa8EJ,XaB66,XaDcI,XaFwM,XaZ0c,XaaZd,K15..,K15z.,K1901,K1902,K1903,K190z,X30NV,X30NY,X30NZ,X30Na,X30PX,X30Pe,XE0e0,XE0e1	81902
Kidney stone	592	N20	K1006,K12..,K120.,K1200,K120z,K121.,K122.,K12z.,Kyu3.,K1006,K12..,K120.,K1200,K120z,K121.,K122.,K12z.	1AC0.,4G4..,4G4..,4G41.,4G43.,4G44.,4G44.,4G6..,K10..,K100.,K1006,K1006,K1020,K1021,K12..,K120.,K1200,K1200,K120z,K121.,K121.,K12z.,K140.,K1400,X30Mv,X30Mv,X30N3,X30N3,X30Pl,X30Pl,X30Pm,X30Pm,X30Pn,X30Po,X30Po,X30Pp,X30Pp,X30Pq,X30Pq,X30Pr,X30Pr,X30Pr,X30Ps,XE0dj,XE0dk,XE0dk,XE26Q,XE2bJ,XM06H,XM06I,XM06J,XM14o,XM1V2,XM1V2,Xa85J,Xa85J,XaA07,XaA07,1AC0.,C341.,K1006,K1200,K120z,K121.,K12z.,K1A..,X30Ke,X30Kf,X30Kg,X30Pl,X30Pl,X30Pm,X30Pm,X30Pn,X30Po,X30Po,X30Pp,X30Pq,X30Pq,X30Pr,X30Pr,X30Ps,X30Pt,XE0dj,XE0dj,XE0dk,XE11c,XM06H,XM14o	201620
Forearm fracture	813	S52	S2240,S2250,S23..,S230.,S2300,S2301,S2302,S2303,S2304,S2305,S2306,S2307,S2308,S2309,S230A,S230B,S230z,S231.,S2310,S2311,S2312,S2313,S2314,S2315,S2316,S2317,S2318,S2319,S231A,S231B,S	S2240,S2250,S23..,S23..,S230.,S2300,S2300,S2300,S2300,S2300,S2300,S2301,S2302,S2303,S2304,S2305,S2306,S2307,S2308,S2309,S230A,S230A,S230B,S230z,S231.,S2310,S2311,S2312,S2313,S2314,S2315,S2316,S2317,S2318,S2319,S231A,S231B,S231z,S232.,S2320,S2321,S2322,S2323,S232z,S233.,S2330,S2331,S2332,S2333	4278672

© 2025 Wang J et al. *JAMA Network Open*.

			231z,S232.,S2320,S2321,S2322,S2323,S232z,S233.,S2330,S2331,S2332,S2333,S233z,S234.,S2340,S2341,S2342,S2343,S2344,S2345,S2346,S2347,S2348,S2349,S234A,S234B,S234C,S234D,S234E,S234F,S234G,S234z,S235.,S2350,S2351,S2352,S2353,S2354,S2355,S2356,S2357,S2358,S2359,S235A,S235B,S235C,S235D,S235E,S235F,S235z,S236.,S237.,S238.,S239.,S23A.,S23B.,S23C.,S23x.,S23x0,S23x1,S23x2,S23x3,S23xz,S23y.,S23y0,S23y1,S23y2,S23y3,S23yz,S23z.,S293.,S4B..,S4B0.,S4B00,S4B01,S4B1.,S4B10,S4B11,S4B2.,S4B20,S4B21,S4B3.,S4B30,S4B31,S4C00,S4C01,S4C10,S4C11,S4C20,S4C21,S4C30,S4C31,Syu53,Syu54,S23..,S230.,S2300,S2301,S2302,S2303,S2304,S2305,S2306,S2307,S2308,S2309,S230A,S230B,S230z,S231.,S2310,S2311,S2312,S2313,S2314,S2315,S2316,S2317,S2318,S2319,S231A,S231B,S231z,S232.,S2320,S2321,S2322,S2323,S232z,S233.,S2330,S2331,S2332,S2333,S233z,S234.,S2340,S23	,S233z,S234.,S2340,S2341,S2342,S2343,S2344,S2345,S2346,S2347,S2348,S2349,S234A,S234B,S234C,S234D,S234E,S234z,S235.,S2350,S2351,S2352,S2353,S2354,S2355,S2356,S2357,S2358,S2359,S235A,S235B,S235C,S235D,S235E,S235z,S23x.,S23x0,S23x0,S23x1,S23x2,S23x3,S23x3,S23x3,S23xz,S23y.,S23y.,S23y.,S23y0,S23y1,S23y2,S23y3,S23y3,S23y3,S23yz,S23z.,S293.,S4B..,S4B0.,S4B00,S4B01,S4B1.,S4B10,S4B11,S4B2.,S4B20,S4B21,S4B3.,S4B30,S4B31,S4C10,S4C11,S4C30,S4C31,Syu53,Syu54,XA001,XA0E2,XA0E3,XA0E4,XA0E4,XA0E5,XA0EQ,XA0ET,XA0EU,XA0GV,XA0GV,XA0GV,XA0GV,XA0GV,XA0GV,XA0GV,XA0GW,XA0GW,XA0GX,XA0GY,XA0GZ,XA0Ga,XA0Ga,XA0Gb,XA0Gc,XA0Gd,XA0Ge,XA0Gf,XA0Gg,XA0Gg,XA0Gg,XA0Gg,XA0Gg,XA0Gg,XA0Gh,XA0Gh,XA0Gi,XA0Gj,XA0Gj,XA0Gk,XA0Gl,XA0Gm,XA0Gn,XE1ks,XE1ks,XE1kt,XE1ku,XE1kv,XE1kw,XE1kx,XE1ky,XE1kz,XE1l0,XM00X,XSBQ2,Xa1mj,Xa1mj,Xa1mk,Xa1ml,Xa1mn,Xa2Ci,Xa8Hw,Xa8ID,XaB0Y,XaBDD,XaBDE,XaBsO,XaIS8,XaXeR,S230.,S2300,S2301,S2302,S2303,S2304,S2305,S2306,S2307,S2308,S2309,S230A,S230B,S230z,S231.,S2310,S2311,S2312,S2313,S2314,S2315,S2316,S2317,S2318,S2319,S231A,S231B,S231z,S232.,S2320,S2321,S2322,S2323,S232z,S233.,S2330,S2331,S2332,S2333,S233z,S2340,S2343,S2344,S2345,S2346,S2347,S2348,S2349,S234A,S234B,S234C,S234D,S234E,S234z,S2350,S2353,S2354,S2355,S2356,S2357,S2358,S2359,S235A,S235B,S235C,S235D,S235E,S235z	

© 2025 Wang J et al. *JAMA Network Open*.

			41,S2342,S2343,S2344,S2345,S2346,S2347,S2348,S2349,S234A,S234B,S234C,S234D,S234E,S234F,S234z,S235.,S2350,S2351,S2352,S2353,S2354,S2355,S2356,S2357,S2358,S2359,S235A,S235B,S235C,S235D,S235E,S235F,S235z,S236.,S237.,S238.,S239.,S23A.,S23B.,S23C.,S23x.,S23x0,S23x1,S23x2,S23x3,S23xz,S23y.,S23y0,S23y1,S23y2,S23y3,S23yz,S23z.,S4C0.,S4C00,S4C10,S4C20,S4C30	,S23x.,S23x0,S23x3,S23xz,S23y.,S23y0,S23y1,S23y2,S23y3,S23yz,S23z.,S23z.,S4C0.,S4C00,S4C10,S4C20,S4C30,Syu53,XA0Dx,XA0E3,XA0E3,XA0E5,XA0E5,XA0ER,XA0ER,XA0ET,XA0ET,XA0EU,XA0EU,XA0GV,XA0GV,XA0GW,XA0GW,XA0GX,XA0GX,XA0GY,XA0GY,XA0GZ,XA0GZ,XA0Ga,XA0Ga,XA0Gb,XA0Gb,XA0Gc,XA0Gc,XA0Gd,XA0Gd,XA0Ge,XA0Ge,XA0Gf,XA0Gf,XA0Gg,XA0Gg,XA0Gh,XA0Gh,XA0Gi,XA0Gi,XA0Gj,XA0Gj,XA0Gk,XA0Gk,XA0Gl,XA0Gl,XA0Gm,XA0Gm,XA0Gn,XA0Gn,XE1ks,XE1ks,XE1kt,XE1ku,XE1kv,XE1kw,XE1kx,XE1ky,XE1kz,XE1l0,XM00X,XM00X,Xa1mj,Xa1mj,Xa1mk,Xa1mk,Xa1ml,Xa1ml,Xa1mn,Xa1mn	
GI bleeding	578	K92.2	J68..,J68z.,J68z0,J68z1,J68z2,J68zz,J68..,J680.,J681.,J68z.,J68z0,J68z1,J68z2,J68zz	J68..,J68z.,J68z0,J68z1,J68zz,X30Be,X30Bi,X30Bj,XE0bJ,Xa00e,XaB3J,XaB3K,J68..,J68..,J680.,J68z0,J68z1,J68zz,X30Be,X30Be,X30Bi,X76fJ,XE0bI,XE0bJ,XE0rA,Xa00e	

Abbreviations: T2DM, type 2 diabetes mellitus; UTI, urinary tract infection; GI bleeding, gastrointestinal bleeding.

© 2025 Wang J et al. *JAMA Network Open*.

eTable 2. Associations of exposures with dementia incidence

	UK Biobank				All of Us			
	Model1		Model2		Model1		Model2	
	HR	95% CI	HR	95% CI	HR	95% CI	HR	95% CI
T2DM								
Overall	2.00	1.85 to 2.15	1.94	1.79 to 2.10	2.39	2.10 to 2.73	2.43	2.11 to 2.79
0-1 year	1.56	1.06 to 2.30	1.43	0.95 to 2.13	3.36	2.37 to 4.76	3.31	2.28 to 4.80
1-5 years	1.65	1.37 to 1.98	1.62	1.34 to 1.95	2.31	1.90 to 2.81	2.29	1.86 to 2.82
5-10 years	1.67	1.44 to 1.94	1.64	1.41 to 1.91	1.98	1.61 to 2.44	2.02	1.63 to 2.51
>10 years	2.32	2.11 to 2.55	2.25	2.04 to 2.48	2.77	2.29 to 3.35	2.88	2.37 to 3.51
Depression								
Overall	2.38	2.21 to 2.57	2.30	2.13 to 2.48	3.51	3.08 to 3.99	3.51	3.08 to 4.01
0-1 year	6.15	4.91 to 7.70	6.02	4.81 to 7.54	7.67	5.92 to 9.93	7.56	5.77 to 9.90
1-5 years	4.06	3.52 to 4.69	3.89	3.37 to 4.50	3.51	2.90 to 4.24	3.43	2.82 to 4.17
5-10 years	3.04	2.64 to 3.51	2.94	2.55 to 3.39	3.35	2.76 to 4.08	3.41	2.79 to 4.16
>10 years	1.70	1.54 to 1.89	1.63	1.47 to 1.81	3.27	2.70 to 3.97	3.37	2.77 to 4.09
Hypertension								
Overall	1.50	1.41 to 1.60	1.44	1.35 to 1.54	2.93	2.49 to 3.45	2.94	2.49 to 3.48
0-1 year	1.56	1.23 to 1.99	1.52	1.19 to 1.94	3.35	2.39 to 4.70	3.27	2.30 to 4.66
1-5 years	1.50	1.33 to 1.70	1.45	1.28 to 1.64	3.00	2.46 to 3.67	2.89	2.35 to 3.56
5-10 years	1.44	1.30 to 1.60	1.39	1.25 to 1.54	2.62	2.14 to 3.20	2.63	2.14 to 3.24
>10 years	1.59	1.48 to 1.71	1.52	1.42 to 1.64	3.37	2.79 to 4.08	3.52	2.90 to 4.28
UTI								
Overall	1.88	1.76 to 2.02	1.85	1.73 to 1.98	2.44	2.13 to 2.79	2.32	2.02 to 2.67
0-1 year	6.73	5.75 to 7.87	6.52	5.57 to 7.65	5.11	3.85 to 6.77	4.87	3.63 to 6.55
1-5 years	2.92	2.59 to 3.28	2.83	2.51 to 3.19	2.59	2.11 to 3.18	2.28	1.84 to 2.84

© 2025 Wang J et al. *JAMA Network Open*.

5-10 years	1.49	1.31 to 1.70	1.46	1.28 to 1.66	2.18	1.74 to 2.72	2.17	1.73 to 2.71
>10 years	1.49	1.36 to 1.63	1.48	1.34 to 1.62	2.11	1.70 to 2.61	2.10	1.69 to 2.62
Kidney stone								
Overall	1.21	1.02 to 1.43	1.18	1.00 to 1.40	1.86	1.53 to 2.25	1.86	1.53 to 2.27
0-1 year	1.35	0.60 to 3.00	1.33	0.60 to 2.97	3.15	1.97 to 5.02	2.82	1.69 to 4.71
1-5 years	1.34	0.89 to 2.02	1.34	0.89 to 2.03	1.62	1.16 to 2.25	1.65	1.18 to 2.30
5-10 years	1.36	0.95 to 1.94	1.35	0.94 to 1.93	2.13	1.54 to 2.93	2.16	1.56 to 2.99
>10 years	1.12	0.90 to 1.40	1.08	0.87 to 1.36	1.50	1.02 to 2.22	1.55	1.05 to 2.29
Forearm fracture								
Overall	1.19	1.07 to 1.33	1.20	1.08 to 1.34	1.97	1.50 to 2.59	2.00	1.52 to 2.63
0-1 year	1.78	1.03 to 3.08	1.84	1.06 to 3.17	2.64	1.26 to 5.57	2.76	1.31 to 5.82
1-5 years	1.64	1.27 to 2.13	1.67	1.29 to 2.17	1.41	0.83 to 2.39	1.46	0.86 to 2.48
5-10 years	1.14	0.90 to 1.46	1.17	0.92 to 1.49	2.35	1.47 to 3.75	2.27	1.41 to 3.68
>10 years	1.11	0.96 to 1.27	1.11	0.96 to 1.27	2.08	1.29 to 3.37	2.15	1.33 to 3.48
GI bleeding								
Overall	1.46	1.30 to 1.64	1.41	1.25 to 1.59	2.02	1.71 to 2.38	2.04	1.73 to 2.41
0-1 year	2.15	1.45 to 3.19	2.17	1.46 to 3.22	2.67	1.73 to 4.12	2.56	1.62 to 4.04
1-5 years	1.60	1.27 to 2.01	1.46	1.15 to 1.86	2.16	1.65 to 2.81	2.14	1.63 to 2.81
5-10 years	1.54	1.26 to 1.89	1.52	1.23 to 1.87	2.37	1.79 to 3.13	2.44	1.84 to 3.22
>10 years	1.24	1.03 to 1.50	1.22	1.01 to 1.47	1.47	1.09 to 1.99	1.53	1.13 to 2.07

Abbreviations: T2DM, type 2 diabetes mellitus; UTI, urinary tract infection; GI bleeding, gastrointestinal bleeding.

Note: Model 1 was adjusted for age, sex, race, and the number of APOE-ε4 alleles. Model 2 was further adjusted for education, smoking history, and BMI.

© 2025 Wang J et al. *JAMA Network Open*.

eTable 3. Benchmark estimates derived from existing meta-analyses of cohort studies

Exposure	Study design	Sample size	Source	Meta-analyzed association	EHR-derived association	
					UK Biobank	All of Us
T2DM	Meta-analysis	2,310,330	Chatterjee et al,[2] 2016	RR=1.62 (95% CI= 1.45-1.80) for women; RR=1.58 (95% CI=1.38=1.81) for men	HR=1.95 (95% CI=1.71-2.22) for females; HR=1.95 (95% CI=1.76-2.17) for males	HR=2.45 (95% CI=2.03-2.95) for females; HR=2.42 (95% CI=1.96-2.98) for males
Depression	Meta-analysis	66,532	Cherbuin et al,[3] 2015	HR=1.98 (95% CI=1.50-2.63)	HR=2.30 (95% CI=2.13-2.48)	HR=3.51 (95% CI=3.08-4.01)
Hypertension	Meta-analysis	877,321	Lennon et al,[4] 2019	HR=1.18 (95% CI=1.02-1.35)	HR=1.44 (95% CI=1.35-1.54)	HR=2.94 (95% CI=2.49-3.48)
UTI	Cohort study	417,172	Mawanda et al,[5] 2016	HR=1.13 (95% CI=1.08-1.18)	HR=1.85 (95% CI=1.73-1.98)	HR=2.32 (95% CI=2.02-2.67)
Kidney stone	n/a	n/a	n/a	n/a	HR=1.18 (95% CI=1.00-1.40)	HR=1.86 (95% CI=1.53-2.27)
Forearm fracture	n/a	n/a	n/a	n/a	HR=1.20 (95% CI=1.08-1.34)	HR=1.52 (95% CI=1.52-2.63)
GI bleeding	n/a	n/a	n/a	n/a	HR=1.41 (95% CI=1.25-1.59)	HR=2.04 (95% CI=1.73-2.41)

Abbreviations: T2DM, type 2 diabetes mellitus; UTI, urinary tract infection; GI bleeding, gastrointestinal bleeding; EHR, electronic health record.

Note: EHR derived associations represent the overall hazard ratios estimated in the full model in our primary analysis.

© 2025 Wang J et al. *JAMA Network Open*.

eTable 4. Pooled detection bias estimates in random-effects models

	Point estimate (95% CI)	
Each exposure	UK Biobank	All of Us
T2DM	0.74 (0.49 to 1.11)	1.36 (0.92 to 2.03)
Depression	2.62 (2.07 to 3.32)	2.15 (1.59 to 2.90)
Hypertension	1.06 (0.82 to 1.36)	1.11 (0.75 to 1.64)
UTI	3.53 (2.97 to 4.20)	2.10 (1.51 to 2.91)
Kidney stone	1.13 (0.50 to 2.55)	1.52 (0.88 to 2.62)
Forearm fracture	1.53 (0.88 to 2.66)	1.38 (0.62 to 3.06)
GI bleeding	1.54 (1.02 to 2.32)	1.26 (0.77 to 2.04)
Meta-analysis	Combined datasets	
Detection bias	1.60 (1.15 to 2.22)	

Abbreviations: T2DM, type 2 diabetes mellitus; UTI, urinary tract infection; GI bleeding, gastrointestinal bleeding.

Note: Point estimates for detection bias, which were used as inputs for the meta-analysis, were calculated by dividing the HR 0–1 year after the exposure diagnosis by the overall HR, allowing us to mitigate or cancel out the effects of potential residual confounding, true causal relationships, and other study biases commonly encountered in both estimates. We used a random-effects model with inverse variance weights. Size of squares represent the standard error of the point estimate.

© 2025 Wang J et al. *JAMA Network Open*.

eTable 5. Association between type 2 diabetes mellitus and incident dementia, stratified by sex

	UK Biobank				All of Us			
	Model1		Model2		Model1		Model2	
	HR	95% CI	HR	95% CI	HR	95% CI	HR	95% CI
Females	2.05	1.81 to 2.31	1.95	1.71 to 2.22	2.45	2.06 to 2.93	2.45	2.03 to 2.95
Males	1.97	1.79 to 2.17	1.95	1.76 to 2.17	2.32	1.90 to 2.83	2.42	1.96 to 2.98

Note: Model 1 was adjusted for age, sex, race, and the number of APOE-ε4 alleles. Model 2 was further adjusted for education, smoking history, and BMI.

© 2025 Wang J et al. *JAMA Network Open*.

eTable 6. Association between healthcare utilization one year prior to baseline and incident dementia

	UK Biobank		All of Us	
	HR	95% CI	HR	95% CI
Healthcare utilization[a]	1.03	1.02 to 1.03	1.02	1.01 to 1.02

[a] Healthcare utilization was coded linearly.

Note: Healthcare utilization was measured by the number of encounters with the healthcare system recorded in the EHR. Utilization is recorded differently in the two cohorts. In UKB, we tallied the total number of GP and inpatient encounters. In AOU, we counted all-cause patient encounters, including primary care records, inpatient records, procedures, pre-procedural examinations, regular medical examinations, follow-up examinations, screenings, etc.

© 2025 Wang J et al. *JAMA Network Open*.

eFigure 1. Associations of exposures with dementia incidence, stratified by levels of healthcare utilization

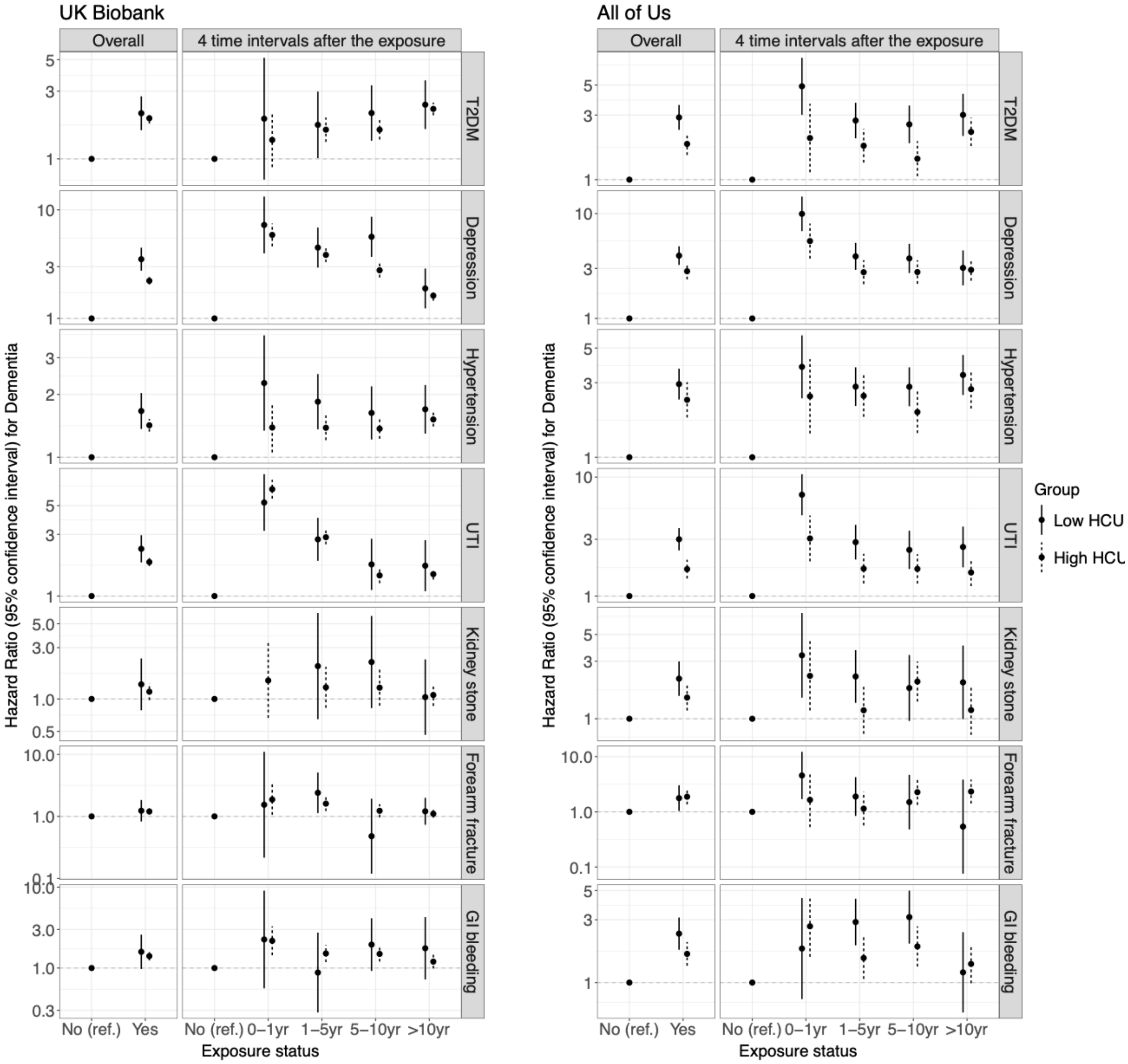

Abbreviations: T2DM, type 2 diabetes mellitus; UTI, urinary tract infection; GI bleeding, gastrointestinal bleeding.

Note: All models were adjusted for age, sex, race, the number of APOE-ε4 alleles, education, smoking history, and BMI.

© 2025 Wang J et al. *JAMA Network Open*.

eFigure 2. Forest plot of random-effects models for the pooled detection bias estimates, stratified by levels of healthcare utilization

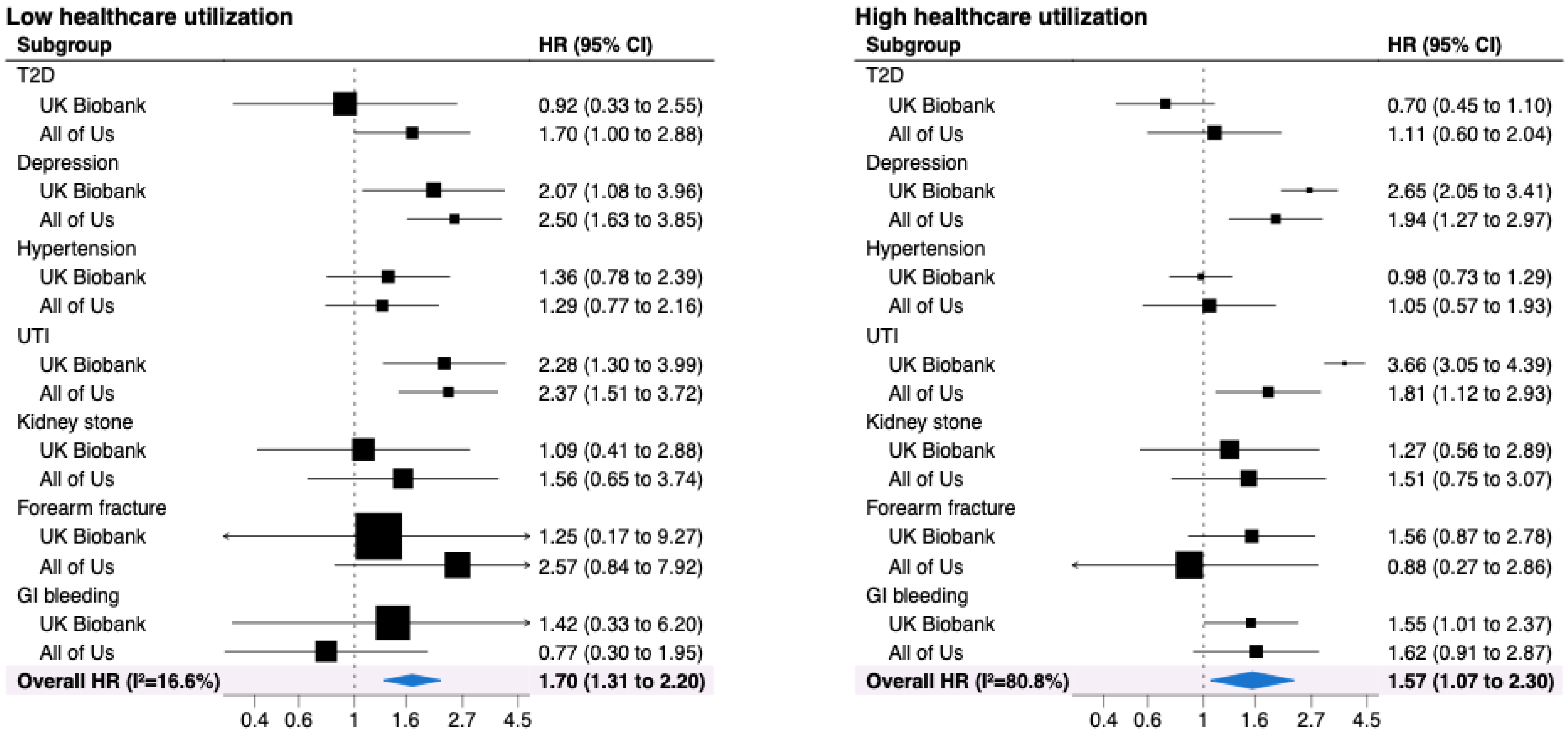

Abbreviations: T2DM, type 2 diabetes mellitus; UTI, urinary tract infection; GI bleeding, gastrointestinal bleeding.
Note: Point estimates for detection bias, which were used as inputs for the meta-analysis, were calculated by dividing the HR 0–1 year after the exposure diagnosis by the overall HR, allowing us to mitigate or cancel out the effects of potential residual confounding, true causal relationships, and other study biases commonly encountered in both estimates. We used a random-effects model with inverse variance weights. Size of squares represent the standard error of the point estimate.

© 2025 Wang J et al. *JAMA Network Open*.

eFigure 3. Associations of exposures with dementia incidence, excluding prevalent exposure cases

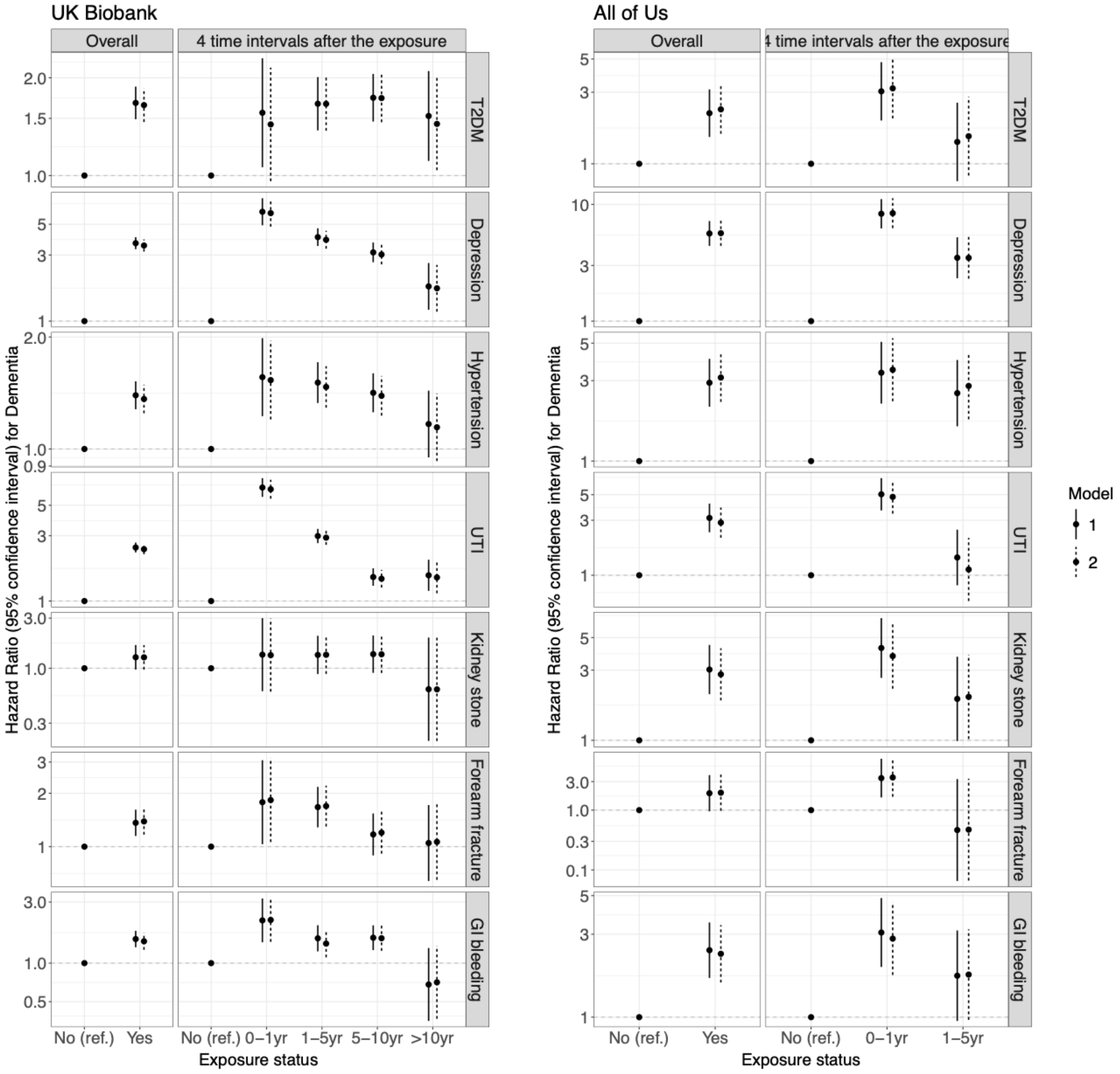

Abbreviations: T2DM, type 2 diabetes mellitus; UTI, urinary tract infection; GI bleeding, gastrointestinal bleeding.

Note: Model 1 was adjusted for age, sex, race, and the number of APOE-ε4 alleles. Model 2 was further adjusted for education, smoking history, and BMI. In this analysis, we excluded participants with prevalent exposure diagnosis before study baseline.

© 2025 Wang J et al. *JAMA Network Open*.

eFigure 4. Forest plot of random-effects models for the pooled detection bias estimates, comparing with estimates 1-2 year after exposure diagnosis

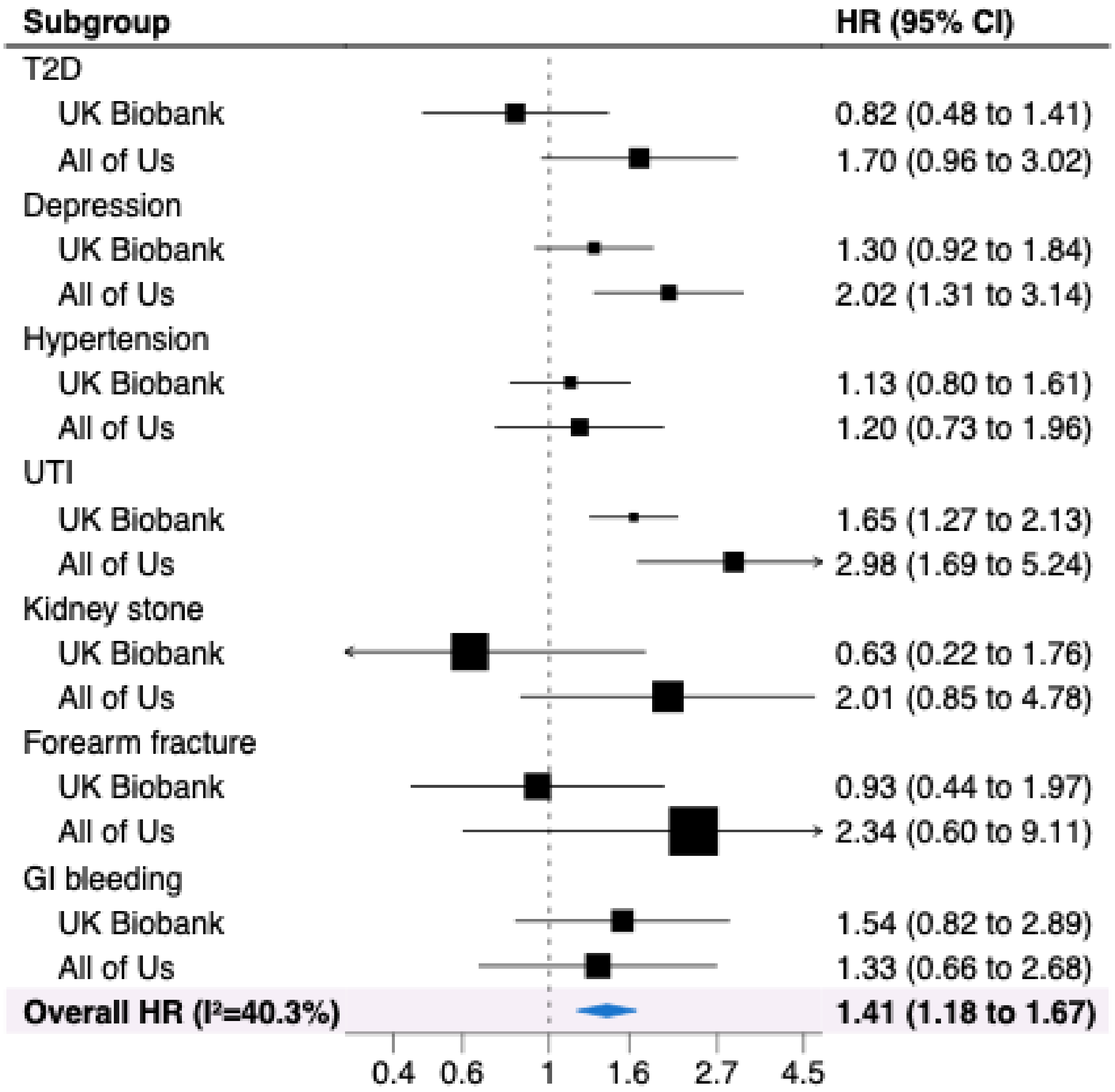

Abbreviations: T2DM, type 2 diabetes mellitus; UTI, urinary tract infection; GI bleeding, gastrointestinal bleeding.
Note: Point estimates for detection bias, which were used as inputs for the meta-analysis, were calculated by dividing the HR 0–1 year after the exposure diagnosis by the HR 0–1 year after the exposure diagnosis. We used a random-effects model with inverse variance weights. Size of squares represent the standard error of the point estimate.

© 2025 Wang J et al. *JAMA Network Open*.

eFigure 5. Forest plot of random-effects models for the pooled detection bias estimates, comparing with estimates from meta-analysis of cohort studies

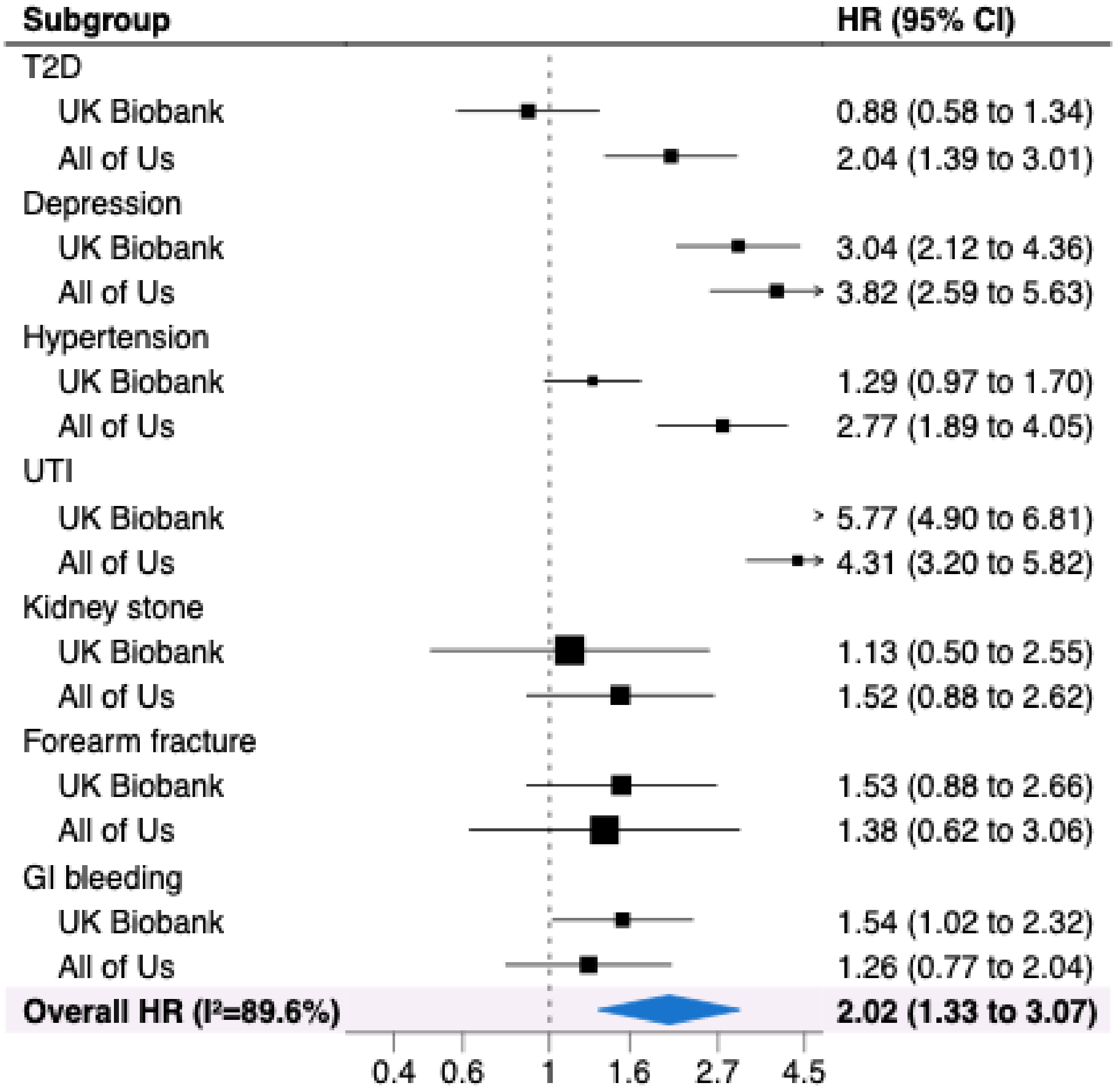

Abbreviations: T2DM, type 2 diabetes mellitus; UTI, urinary tract infection; GI bleeding, gastrointestinal bleeding.

Note: Point estimates for detection bias, which were used as inputs for the meta-analysis, were calculated by dividing the HR 0–1 year after the exposure diagnosis by effect sizes from existing cohort studies or meta-analysis of cohort studies, where available: T2DM,[2] depression,[3] hypertension,[4] and UTI[5]. For kidney stone, forearm fracture, and GI bleeding, there is no existing study, and we divided the HR 0–1 year after the exposure diagnosis by the overall HR in our analysis. Specifically, the comparison HR for T2DM, depression, hypertension, and UTI were 1.62 (95% CI=1.45-1.80), 1.98 (95% CI=1.50-2.63),1.18 (95% CI=1.02-1.35), and 1.13 (95% CI=1.08-1.18), respectively. We used a random-effects model with inverse variance weights. Size of squares represent the standard error of the point estimate.

© 2025 Wang J et al. *JAMA Network Open*.

eReferences:

1. Ramirez AH, Sulieman L, Schlueter DJ, et al. The All of Us Research Program: Data quality, utility, and diversity. *Patterns*. 2022;3(8):100570. doi:10.1016/j.patter.2022.100570

2. Chatterjee S, Peters SAE, Woodward M, et al. Type 2 Diabetes as a Risk Factor for Dementia in Women Compared With Men: A Pooled Analysis of 2.3 Million People Comprising More Than 100,000 Cases of Dementia. *Diabetes Care*. 2016;39(2):300-307. doi:10.2337/dc15-1588

3. Cherbuin N, Kim S, Anstey KJ. Dementia risk estimates associated with measures of depression: a systematic review and meta-analysis. *BMJ Open*. 2015;5(12):e008853. doi:10.1136/bmjopen-2015-008853

4. Lennon MJ, Makkar SR, Crawford JD, Sachdev PS. Midlife Hypertension and Alzheimer's Disease: A Systematic Review and Meta-Analysis. *J Alzheimers Dis*. 2019;71(1):307-316. doi:10.3233/JAD-190474

5. Mawanda F, Wallace RB, McCoy K, Abrams TE. Systemic and localized extra-central nervous system bacterial infections and the risk of dementia among US veterans: A retrospective cohort study. *Alzheimer's & Dementia: Diagnosis, Assessment & Disease Monitoring*. 2016;4:109-117. doi:10.1016/j.dadm.2016.08.004

© 2025 Wang J et al. *JAMA Network Open*.
