# Supplementary material for: Detection Bias in EHR-Based Research on Clinical Exposures and Dementia
Source: JAMA Netw Open. 2025 Apr 23;8(4):e256637. doi: 10.1001/jamanetworkopen.2025.6637 (PMC12019524; doi:10.1001/jamanetworkopen.2025.6637)
Supplement: Supplement 2. — Data Sharing Statement [file jamanetwopen-e256637-s002.pdf]

Data Sharing Statement

Wang. Detection Bias in EHR-Based Research on Clinical Exposures and Dementia. *JAMA Netw Open*. Published April 23, 2025. doi:10.1001/jamanetworkopen.2025.6637

Data
Data available: Yes
Data types: Deidentified participant data
How to access data: All of Us: https://allofus.nih.gov/ UK Biobank: https://www.ukbiobank.ac.uk/
When available: With publication

Supporting Documents
Document types: None

Additional Information
Who can access the data: researchers whose proposed use of the data has been approved
Types of analyses: for a specified purpose
Mechanisms of data availability: after approval of a proposal and with a signed data access agreement
